# Bone-targeting engineered small extracellular vesicles carrying anti-miR-6359-CGGGAGC prevent valproic acid-induced bone loss

**DOI:** 10.1038/s41392-023-01726-8

**Published:** 2024-01-22

**Authors:** Xudong Xie, Peng Cheng, Liangcong Hu, Wu Zhou, Detai Zhang, Samuel Knoedler, Guodong Liu, Yuan Xiong, Hang Xue, Yiqiang Hu, Barbara Kern, Doha Obed, Adriana C. Panayi, Lang Chen, Chenchen Yan, Ze Lin, Guandong Dai, Bobin Mi, Yingze Zhang, Guohui Liu

**Affiliations:** 1 Department of Orthopedics, Union Hospital, Tongji Medical College, Huazhong University of Science and Technology, Wuhan, 430022 China; 2 Hubei Province Key Laboratory of Oral and Maxillofacial Development and Regeneration, Wuhan, 430022 China; 3 Department of Clinical Laboratory, Union Hospital, Tongji Medical College, Huazhong University of Science and Technology, Wuhan, Hubei 430022 P.R. China; 4 Division of Plastic Surgery, Brigham and Women’s Hospital, Harvard Medical School, Boston, MA 02152 USA; 5 Department of Plastic Surgery and Hand Surgery, Klinikum Rechts der Isar, Technical University of Munich, Munich, Germany; 6 Medical Center of Trauma and War Injuries, Daping Hospital, Army Medical University, Chonqing, 400042 China; 7 Department of Plastic Surgery, Campus Charité Mitte|Campus Virchow-Klinikum, Charité-Universitätsmedizin Berlin, corporate member of Freie Universität Berlin, Humboldt-Universität zu Berlin and Berlin Institute of Health, Berlin, Germany; 8 Berlin Institute of Health, Charité - Universitätsmedizin Berlin, Berlin, Germany; 9 Department of Plastic, Aesthetic, Hand and Reconstructive Surgery, Hannover Medical School, Hannover, Germany; 10 Department of Hand, Plastic and Reconstructive Surgery, Microsurgery, Burn Center, BG Trauma Center Ludwigshafen, University of Heidelberg, Ludwig-Guttmann-Strasse 13, 67071 Ludwigshafen/Rhine, Germany; 11 Pingshan District People’s Hospital of Shenzhen, Pingshan General Hospital of Southern Medical University, Shenzhen, Guangdong 518118 China; 12 Department of Orthopaedic Surgery, The Third Hospital of Hebei Medical University, NO.139 Ziqiang Road, Shijiazhuang, 050051 China

**Keywords:** Cell biology, Drug discovery

## Abstract

The clinical role and underlying mechanisms of valproic acid (VPA) on bone homeostasis remain controversial. Herein, we confirmed that VPA treatment was associated with decreased bone mass and bone mineral density (BMD) in both patients and mice. This effect was attributed to VPA-induced elevation in osteoclast formation and activity. Through RNA-sequencing, we observed a significant rise in precursor miR-6359 expression in VPA-treated osteoclast precursors in vitro, and further, a marked upregulation of mature miR-6359 (miR-6359) in vivo was demonstrated using quantitative real-time PCR (qRT-PCR) and miR-6359 fluorescent in situ hybridization (miR-6359-FISH). Specifically, the miR-6359 was predominantly increased in osteoclast precursors and macrophages but not in neutrophils, T lymphocytes, monocytes and bone marrow-derived mesenchymal stem cells (BMSCs) following VPA stimulation, which influenced osteoclast differentiation and bone-resorptive activity. Additionally, VPA-induced miR-6359 enrichment in osteoclast precursors enhanced reactive oxygen species (ROS) production by silencing the SIRT3 protein expression, followed by activation of the MAPK signaling pathway, which enhanced osteoclast formation and activity, thereby accelerating bone loss. Currently, there are no medications that can effectively treat VPA-induced bone loss. Therefore, we constructed engineered small extracellular vesicles (E-sEVs) targeting osteoclast precursors in bone and naturally carrying anti-miR-6359 by introducing of EXOmotif (CGGGAGC) in the 3’-end of the anti-miR-6359 sequence. We confirmed that the E-sEVs exhibited decent bone/osteoclast precursor targeting and exerted protective therapeutic effects on VPA-induced bone loss, but not on ovariectomy (OVX) and glucocorticoid-induced osteoporotic models, deepening our understanding of the underlying mechanism and treatment strategies for VPA-induced bone loss.

## Introduction

Osteoporosis is characterized by systemic skeletal pathology, marked by diminished bone density and a microarchitectural degeneration of bone tissue, leading to heightened fragility and susceptibility to fractures.^1,2^ Factors, such as age, estrogen deficiency, and drugs that disrupt the balance of a coordinated process between bone resorption and bone formation, promote to the development of osteoporosis. Valproic acid (VPA), a branched short-chain fatty acid, is a medication with wide and long-term usage for the management of various types of epilepsy, particularly in patients with generalized epilepsy. However, the risk of osteoporosis has been increasing in patients receiving VPA medication, as evidenced by the decreased bone mineral density (BMD) and accelerated bone-mass loss, which increased the prevalence of osteoporotic fracture, causing significant health complications and health-care costs.^3,4^ Furthermore, there are few guidelines specifically addressing the evaluation of osteoporosis in epilepsy patients undergoing VPA administration. Limited studies have directly examined the impact of conventional treatments, such as antiresorptive or anabolic therapies, on VPA-induced osteopenia or osteoporosis in these patients. Consequently, it is important to investigate the underlying mechanisms and identify potentially effective therapies for addressing this epidemic.

MicroRNAs (miRNAs) are small, single-stranded endogenous non-coding RNAs which are considered to be regulators of diverse biological process, such as cell proliferation, cell cycle, differentiation and organ development.^5,6^ Similarly, miRNAs are also extensively involved in the initiation and progression of osteoporosis by negatively regulating expression of specific genes at post-transcriptional levels.^7,8^ Several miRNAs have been reported to modulate osteoclastogenesis and osteoblastic bone formation, and dysregulation of these miRNAs has been correlated to skeletal diseases marked by bone mass loss. For example, it was found that increased osteoclastic miR-214-3p in elderly women with fractures and in ovariectomized mice was transferred to osteoblasts, thereby inhibiting bone formation.^9^ Enriched miR-92a-1-5p in prostate cancer (PCa)-derived small extracellular vesicles (sEVs) downregulated the expression of type I collagen expression, which promoted osteoclast formation and inhibitied osteogenesis.^10^ Specifically, alterations in miRNAs levels were correlated with fluctuations in levels of bone formation and bone resorption markers as well as BMD.^11,12^ These changes in bone turnover markers were also observed in epilepsy patients on VPA medication,^13,14^ suggesting that miRNAs might participate in the VPA-induced bone loss and osteoporosis.

Current miRNA-based therapies have been applied in various fields, including regenerative medicine and aging-related diseases. Numerous researchers have reported that miR-26a-loaded scaffolding can promote bone regeneration.^15,16^ Similarly, the delivery of anti-miR-221 via hydrogel has shown to effectively improve cartilage repair.^17^ However, local delivery strategies can reduce off-target effects during treatment in the affected location^17^, but they might not be applicable in systemic diseases, such as osteoporosis. Therefore, Hu et al. constructed hybrid nanoparticles using chemokine (C-X-C motif) receptor 4 (CXCR4)^+^ sEVs fused with liposomes carrying antagomiR-188 (hybrid NPs) and demonstrated that they stimulated osteogenesis and suppressed bone marrow-derived mesenchymal stem cells (BMSCs) adipogenesis, thereby alleviating of age-related trabecular bone loss, suggesting that they may be an ideal agent to target the osteogenic lineage cells.^18^ However, nanoparticles targeting osteoclastic lineage cells have been rarely reported and strategies that regulate osteoclast formation and activity are frequently nipped in the bud. In this context, sEVs, characterized by an average diameter of around 100 nanometers, either in their natural form or as carriers for drug payloads, present an active area of exploration as therapeutic agents based on their minimal immune clearance upon exogenous administration, favorable tolerance and low toxicity when repeated in vivo injections.^19^ Further, ligand enrichment on engineered sEVs may improve the targeting to specific cell types. For example, highly expressing THLG on sEVs using the genetic engineering technology, serving as a ligand for HER2, has been shown to augment the binding capacity of sEVs to target cells.^20^ This, in turn, led to the reversal of drug resistance and enhanced the effectiveness of cancer.^20^ CX3C chemokine receptor 1 (CX3CR1), the only receptor for the unique CX3C membrane-anchored chemokine (fractalkine, CX3CL1), is highly expressed in osteoclast precursors, and can be used as a marker for identifying osteoclast precursors in osseous tissue.^21–23^ Therefore, we postulate that highly expressing CX3XL1 on sEVs as a ligand for CX3CR1 on osteoclast precursors could effectively boost the binding ability of sEVs to target osteoclast precursors in skeletal tissue for efficiently transfer specific miRNA to treat the disease.

In previous study, the impact of VPA on bone health has been a subject of controversial, and the specific mechanisms which VPA contributes to disease development remain unclear. In this study, we elucidate the role of VPA in the development of VPA-induced osteopenia or osteoporosis and develop a promising agent designed to effectively reverse VPA-induced osteopenia. First, we collected the dual-energy X-ray absorptiometry (DXA) results and blood samples from patients to explore the effect of VPA on bone health in vivo. Next, we compared the in vivo and in vitro effects of VPA on osteoclast formation and osteogenesis and the captured RNAs are sequenced to probe the underlying mechanisms. Moreover, we established engineered sEVs (E-sEVs) that targeted osteoclast precursors and were naturally and specifically loaded with anti-miR-6359-CGGGAGC to alleviate the VPA-induced osteopenia, indicating that they are a novel treatment strategy for osteopenia or osteoporosis caused by VPA use.

## Results

### Bone loss is increased in patients undergoing VPA treatment and is associated with increased osteoclast formation

**Fig. 1:**
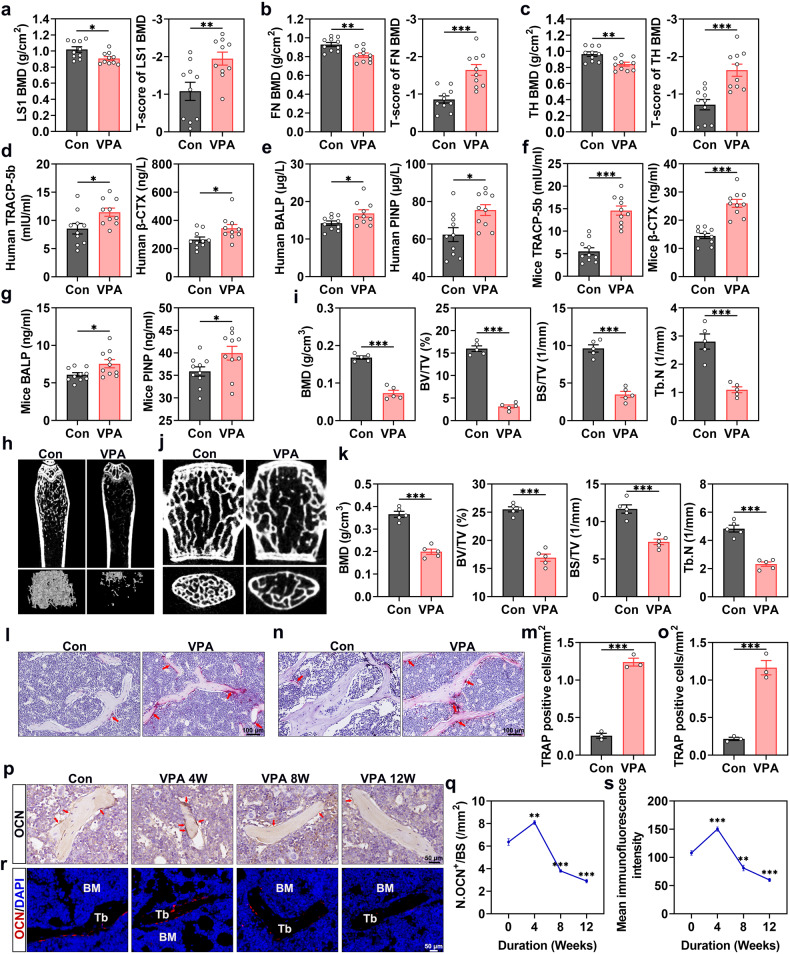
Bone loss is increased in patients undergoing VPA treatment and is associated with increased osteoclast formation. **a** LS1 BMD and the T-score of LS1 BMD (*n* = 10). **b** FN BMD and the T-score of FN BMD (*n* = 10). **c** TH BMD and the T-score of TH BMD (*n* = 10). **d** The TRACP-5b and β-CTX levels in the serum of patients (*n* = 10). **e** The BALP and PINP levels in the serum of patients (*n* = 10). **f** The serum concentrations of TRACP-5b and β-CTX levels in mice (*n* = 10). **g** The BALP and PINP levels in the serum of mice (*n* = 10). **h** Micro-CT images of the femurs in the control and VPA treatment groups (*n* = 5). **i** Quantitative analysis of trabecular bone. **j** Micro-CT images of the lumbar spine in the control and VPA treatment groups (*n* = 5). **k** Illustration of the corresponding morphometric measurements of trabecular bone. **l**, **m** TRAP staining and quantification of the femurs in control and VPA groups (*n* = 3). Scar bar: 100 μm. **n**, **o** TRAP staining and quantification of the lumbar spine in control and VPA groups (*n* = 3). Scar bar: 100 μm. **p**, **q** Representative images of osteocalcin (OCN) immunohistochemical staining and quantification of number of OCN-positive osteoblasts in distal femurs (*n* = 3). Red arrows represent OCN-positive cells. Scar bar: 50 μm. **r**, **s** Immunofluorescence staining for OCN and quantitative analysis of the images (*n* = 3). Scar bar: 50 μm. Data are presented as the mean ± SEM (*n* ≥ 3) (*p < 0.05; **p < 0.01; ***p < 0.001)

DXA analysis was performed on the included patients to determine their BMD and investigate the effect of VPA on bone health. According to the results, VPA treatment was associated with reduced BMD at the lumbar spine 1 (LS1; Fig. 1a), femoral neck (FN; Fig. 1b), and total hip (TH; Fig. 1c). Accordingly, patients undergoing VPA therapy exhibited a significant reduction in mean BMD T-score at LS1, FN, and TH (Fig. 1a–c), which was in line with previous studies.^24,25^ Reduced BMD would be due to an imbalance between osteoblast and osteoclast activity.^26^ Furthermore, Enzyme‐linked immunosorbent assay (ELISA) was performed to determine to the circulating levels of markers for bone resorption, including tartrate-resistant acid phosphatase isoform 5b (TRACP-5b) and beta-C-terminal telopeptides of type I collagen (β-CTX), and bone formation, including procollagen type I N-terminal propeptide (PINP) and bone alkaline phosphatase (BALP). Serum TRACP-5b and β-CTX concentrations were increased in patients undergoing VPA treatment (Fig. 1d), as were serum PINP and BALP levels (Fig. 1e).

Subsequently, we used four-week VPA-treated mice model to mimic patients on VPA medications. Blood samples were obtained from mice with or without VPA treatment to determine the levels of bone turnover markers. Mice undergoing VPA treatment showed increased serum TRACP-5b and β-CTX levels (Fig.1f). Additionally, serum PINP and BALP levels were upregulated after VPA exposure (Fig. 1g), which was concordant with our clinical findings. Furthermore, the femurs and lumbar spine were collected for microcomputed tomography (micro-CT) analysis of osseous tissue microstructural changes. The results indicated low bone mass and trabecular micro-architecture deterioration in the VPA-treated group (Fig. 1h, j), which was corroborated by morphometric analyses of trabecular indices, including BMD, bone volume/total volume (BV/TV), bone surface area/total value (BS/TV), and trabecular number (Tb. N) (Fig. 1i, k). Similar results were obtained in female mice (Supplementary Fig. 1a–c). These findings indicate that VPA treatment could increase bone loss by accelerating bone tissue turnover, with elevated osteoclastic formation and activity highly likely to be the primary cause.

The distal femur and lumbar spine sections were stained with tartrate-resistant acid phosphatase (TRAP) to determine whether VPA-induced bone loss was lined to elevated osteoclast activity. Compared to the control group, VPA treatment displayed a higher number of mature osteoclasts (Fig. 1l–o). To elucidate this phenomenon, we isolated osteoclast precursors from femurs and tibias of mice with or without VPA treatment, and the differentiation capacity was then evaluated by TRAP staining. In line with our previous findings, we observed a higher osteoclast population with larger sizes after VPA treatment (Supplementary Fig. 1d, e). We also explored the effect of VPA on osteogenesis through the immunohistochemical and immunofluorescence staining of osteocalcin (OCN), a bone formation marker. We observed more OCN-positive cells after four weeks of VPA treatment in vivo, which was consistent with the ELISA results (Fig. 1p–s). Interestingly, compared to the control group, the OCN level decreased after 8 and 12 weeks of VPA treatment (Fig. 1p–s). In vitro VPA treatment experiments did not significantly enhance alkaline phosphatase (ALP) and alizarin red S (ARS) activity (Supplementary Fig. 1f-i), implying that there was no direct evidence of in vitro VPA-induced bone formation in our investigation and the increase in OCN level in vivo might be stimulated by increased bone resorption. These findings collectively suggest that VPA-induced bone loss may be attributed, at least in part, to elevated osteoclast formation and activity.

### miR-6359 is enriched in VPA-treated osteoclast precursors and is responsible for osteoclast differentiation

**Fig. 2:**
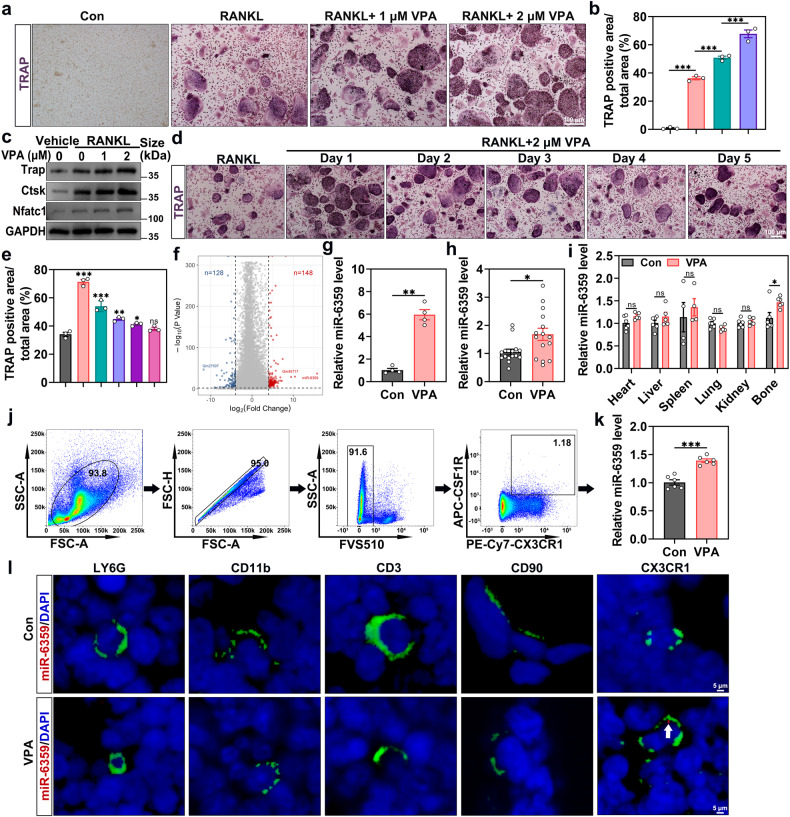
miR-6359 is enriched in VPA-treated osteoclast precursors and is responsible for osteoclast differentiation. **a** Representative images displaying the TRAP-positive multinuclear cells (*n* = 3). Scar bar: 100 μm. **b** Quantitative analysis of TRAP-positive area/total area (%). **c** Western blot analysis of the impact of VPA treatment on Nfatc1, Ctsk and Trap protein levels. **d** TRAP staining of osteoclast precursors treated with VPA at different stages of osteoclastogenesis (*n* = 3). Scar bar: 100 μm. **e** The area of TRAP-positive cells/total area (%) was counted. **f** Volcano plot showing genes or miRNAs with a cut-off fold-change of ≥ 4 or ≤ −4 and a p value of < 0.01. **g** qRT-PCR detection of the relative abundance of miR-6359 in osteoclast precursors treated with or without VPA (*n* = 4). **h** Relative abundance of miR-6359 in the serum of mice treated with or without VPA, as determined by qRT-PCR analysis (*n* = 15). **i** qRT-PCR detection of miR-6359 expression level in various tissues, such as heart, liver, spleen, lung, kidney and bone following VPA treatment (*n* = 4, 5 or 6). **j**, **k** CX3CR1^+^, Csf1R^+^ osteoclast precursors were identified (**j**) and the miR-6359 level was measured through qRT-PCR analysis (**k**) (*n* = 6). **l** miR-6359-FISH and immunofluorescence for LY6G, CD11b, CD3, CD90 and CX3CR1 were performed. Scar bar: 5 μm. Data are presented as the mean ± SEM (n ≥ 3) (*p < 0.05; **p < 0.01; ***p < 0.001)

To clarify the role of VPA in osteoclastogenesis, cell counting kit 8 (CCK8) assays were carried out prior to the in vitro study. The results showed that VPA concentrations of ≤ 2 μM had no effect on the viability of osteoclast precursors (Supplementary Fig. 2a). As a result, we used osteoclast precursors as a standard cellular osteoclastogenesis model to investigate the effects of VPA on osteoclast formation in vitro. Osteoclastogenesis was assessed by TRAP staining after treating the osteoclast precursors with 0, 1, or 2 μM VPA along with 30 ng/ml microglia colony stimulating factor (M-CSF) and 50 ng/ml receptor activator of nuclear factor kappa-Β ligand (RANKL) for five days. The population and size of TRAP-positive cells in VPA-treated groups grew dramatically in a dose-dependent manner (Fig. 2a, b). Western blot analysis revealed that osteoclastogenesis-related proteins (Nfatc1, Ctsk and Trap) expression levels were elevated in the vehicle-treated group upon M-CSF and RANKL stimulation. Furthermore, VPA treatment significantly fostered the expression of these proteins (Fig. 2c), which accorded with the qRT-PCR analysis results (Supplementary Fig. 2b).

Subsequently, we examined the effects of VPA on RANKL-induced osteoclastogenesis by applying the treatment at different time points from day 1 to 5 following RANKL stimulation to determine whether VPA promotes early or late osteoclastogenesis. We observed that VPA treatment significantly promoted RANKL-induced osteoclastogenesis, especially the first 2 days (Fig. 2d, e). To define the expression profile associated with VPA treatment, we conducted an RNA sequencing analysis of mRNAs or miRNAs from osteoclast precursors. We identified 21433 genes, of which 276 were differentially expressed (absolute fold change ≥ 4 or ≤ −4; *p* < 0.01) in osteoclast precursors after VPA treatment. Compared to the control group, 148 and 128 genes were significantly upregulated and downregulated in osteoclast precursors post-VPA treatment, respectively. Among them, precursor miR-6359, Gm45717 and Gm27597 were the most significantly expressed genes (Fig. 2f). Given that Gm45717 and Gm27597 are genes of unknown function, we decided to further investigate miR-6359.

We employed qRT-PCR analysis to determine whether mature miR-6359 (miR-6359) expression changed in VPA-treated osteoclast precursors. As demonstrated in Fig. 2g, miR-6359 was enriched in VPA-treated cells. Furthermore, miR-6359 level in mice serum markedly increased after VPA treatment (Fig. 2h). Subsequently, to explore miR-6359 function in osteoclast precursors, we used specific agomiRs or antagomiRs to upregulate or knockdown it, respectively. Flow cytometry analysis indicated that compared to the negative vehicle-treated osteoclast precursors, incubation with the agomiR-Cy3 indicator for 8 hours increased the population of Cy3 fluorescent-positive osteoclast precursors (Supplementary Fig. 2c, d), implying that agomiRs or antagomiRs infiltrated the osteoclast precursors. As shown by TRAP staining and qRT-PCR analysis (Supplementary Fig. 2e–g), miR-6359 overexpression via agomiR-6359 further enhanced the capacity of VPA to induce osteoclast formation and promote Nfatc1, Ctsk, and Trap expression in osteoclast precursors under osteoclastogenesis. On the other hand, antagomiR-6359 noticeably suppressed the pro-osteoclastic effects of VPA by inhibiting miR-6359 (Supplementary Fig. 2e–g). Consistent with the osteoclastic formation results, although agomiR-6359-pretreated osteoclast precursors induced more resorption pits and a larger resorption area than the VPA-treated osteoclast precursors, antagomiR-6359 could inhibit the VPA effect on bone resorption (Supplementary Fig. 2h, i). These findings suggest that miR-6359 mediates the VPA-induced promotion of osteoclastic formation and activity.

Subsequently, after VPA treatment, we first performed qRT-PCR analysis for the heart, liver, spleen, lung, kidney and bone tissues to explore the expression of miR-6359 in various tissues. The results revealed a predominant increase in miR-6359 expression level in bone, with no difference in the miR-6359 expression level in other tissues after VPA treatment compared to controls (Fig. 2i). Following VPA stimulation, the miR-6359 expression levels in various cells in bone was investigated further. Specifically, bone marrow from the femurs and tibias were collected for flow cytometry and cell sorting after treating mice with VPA for four weeks, in which neutrophils, T lymphocytes, macrophages, monocytes, BMSCs and osteoclast precursors were sorted for qRT-PCR analysis. Different miR-6359 expression levels were detected in macrophages and osteoclast precursors. However, miR-6359 expression levels in neutrophils, T lymphocytes, monocytes and BMSCs in bone were comparable to those in controls (Fig. 2j, k, Supplementary Fig. 3a–j). Additionally, fluorescence in situ hybridization (FISH) analysis of miR-6359 was performed in femur sections, and CX3CR1^+^ cells (osteoclast precursors) with intracellular red fluorescent signals were observed after VPA treatment (Fig. 2l). Conversely, there were fewer red fluorescent signals in Ly6G^+^ cells (neutrophils), CD11b^+^ cells (monocytes and macrophages), CD3^+^ cells (T lymphocytes) and CD90^+^ cells (BMSCs) (Fig. 2l). Together, VPA-induced increase in miR-6359 level may be cell-type specific, and it was mainly increased in the osteoclast precursors.

### SIRT3 is a target of miR-6359 during osteoclastogenesis

**Fig. 3:**
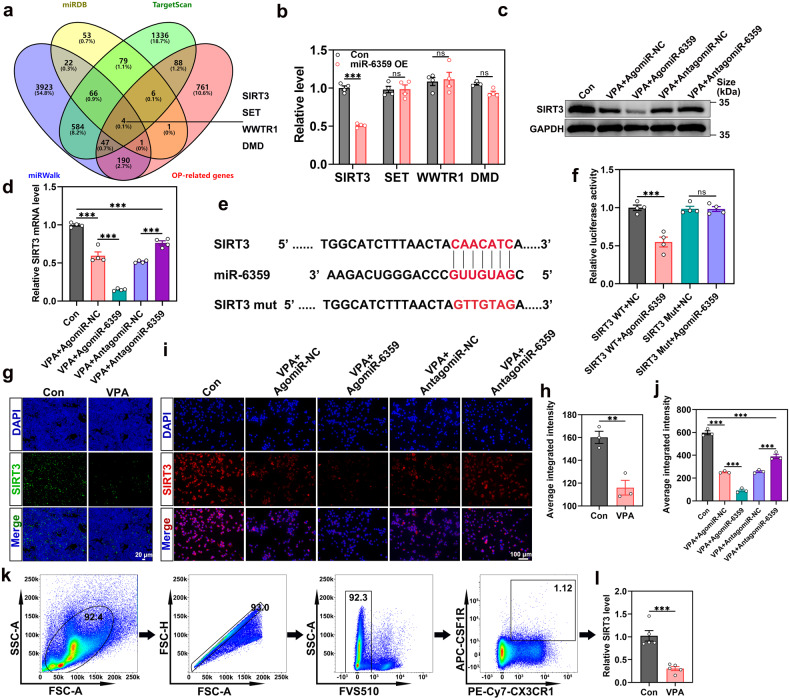
SIRT3 is a target of miR-6359 during osteoclastogenesis. **a** Venn diagram showing the miR-6359 targets from miRWalk, TargetScan, miRDB database and OP-related genes. **b** qRT-PCR analysis of suspected miR-6359 targets (SIRT3, SET, WWTR1 and DMD) (*n* = 3 or 4). **c** Western blot quantification of SIRT3 expression level in osteoclast precursors with indicated treatments. **d** qRT-PCR analysis of SIRT3 expression level in osteoclast precursors with different treatment (*n* = 4). **e** Alignment between miR-6359 with the 3’ UTR of SIRT3 showing potential binding sites. **f** SIRT3 wild-type and mutant cell luciferase activity after transfection with agomiR-6359 or agomiR-NC (*n* = 4). **g**, **h** Immunofluorescence staining and quantitative analysis of SIRT3 in femoral sections of mice treated with vehicle or VPA (*n* = 3). Scar bar: 20 μm. **i**, **j** Immunofluorescence staining and quantification of SIRT3 in osteoclast precursors with indicated treatments (*n* = 3). Scar bar: 100 μm. **k**, **l** CX3CR1^+^, Csf1R^+^ osteoclast precursors were sorted by flow cytometry (**k**) and qRT-PCR analysis was used to detect the expression level of SIRT3 (**l**) (*n* = 5). Data are presented as the mean ± SEM (n ≥ 3) (*p < 0.05; **p < 0.01; ***p < 0.001)

To investigate novel downstream molecules, three bioinformatics databases (miRWalk, TargetScan, and miRDB) and osteoporosis-related genes (OP-related genes) were used to determine the potential miR-6359 targets, and SIRT3, SET, WWTR1 and DMD were selected (Fig. 3a). The changed expression of the four genes in osteoclast precursors transfected with agomiR-6359 or agomiR-NC was then used to evaluate through qRT-PCR analysis. We observed that SIRT3 expression, which negatively regulates osteoclast formation^27^, was remarkably suppressed upon agomiR-6359 overexpression in osteoclast precursors (Fig. 3b). In other words, transfection with agomiR-6359 lowered the endogenous SIRT3 protein expression in osteoclast precursors. On the other hand, miR-6359 inhibition upon antagomiR-6359 transfection significantly elevated the SIRT3 protein level (Fig. 3c), which was also confirmed by qRT-PCR analysis (Fig. 3d). The SIRT3 3’ UTR sequence was screened in the TargetScan database (V 7.2, https://www.targetscan.org/vert_72/) to identify the miR-6359 binding site, in which the identical sequence in the miR-6359 seed region binds to SIRT3 via seven base pair (Fig. 3e). Subsequently, we cloned the 3’UTRs of the mutative target in a dual luciferase assay system to further determine whether miR-6359 targeted SIRT3 directly. In reporter assays, agomiR-6359 alone considerably reduced luciferase activity, whereas binding site mutations exerted no discernible effect on luciferase activity (Fig. 3f). Furthermore, consistent with the above cell experiment results, immunofluorescence staining for distal femurs from mice with or without VPA treatment showed a significantly lower SIRT3 expression in the VPA treatment group (Fig. 3g, h). Additionally, immunofluorescence staining for SIRT3 in osteoclast precursors revealed that treatment with VPA in combination with agomiR-6359 significantly lowered SIRT3 protein expression compared to treated with VPA and agomiR-NC, whereas antagomiR-6359 significantly blocked the VPA-mediated SIRT3 downregulation (Fig. 3i, j). Meanwhile, the qRT-PCR analysis of sorted osteoclast precursors obtained from femurs and tibias demonstrated diminished SIRT3 levels in osteoclast precursors following VPA stimulation (Fig. 3k, l), which was consistent with immunofluorescence staining results. Notably, the osteoclastogenesis effect of miR-6359 could be attributed to SIRT3 protein downregulation, indicating that SIRT3 might be the downstream miR-6359 target, which negatively regulates osteoclast formation.

### SIRT3 inhibition promotes ROS production in osteoclast precursors and osteoclast formation via the MAPK signaling pathway

**Fig. 4:**
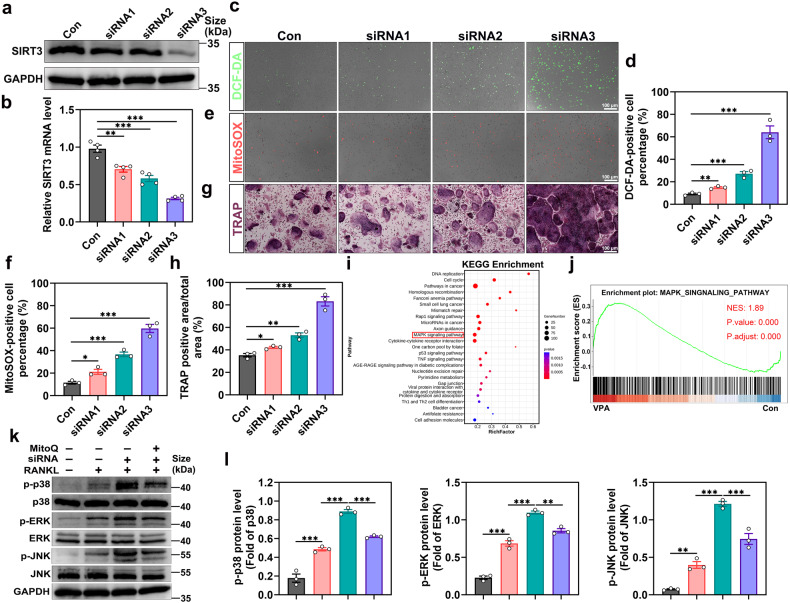
SIRT3 inhibition promotes ROS production in osteoclast precursors and osteoclast formation via the MAPK signaling pathway. **a** Western blot quantification of SIRT3 expression in osteoclast precursors treated with SIRT3 siRNAs or negative control. **b** qRT-PCR analysis of SIRT3 expression in osteoclast precursors transfected with SIRT3 siRNAs or negative control (*n* = 4). **c** Approximately 4 × 10^4^ osteoclast precursors were seeded in 24-well plates and grown overnight. After 24 h of incubation with SIRT3 siRNAs or negative control in the presence of 30 ng/ml M-CSF and 50 ng/ml RANKL, DCFH-DA staining was performed (*n* = 3). Scar bar: 100 μm. **d** Quantification of DCF-DA-positive cell percentage in (**c**). **e** Measurement of mitochondrial ROS using MitoSOX Red (*n* = 3). Scar bar: 100 μm. **f** Quantification of MitoSOX-positive cell percentage in (**e**). **g** TRAP-positive cells images after transfection (*n* = 3). Scar bar: 100 μm. **h** Quantitative analysis of TRAP-positive area/total area (%). **i** KEGG pathway enrichment analysis of differentially expressed genes (foldchange ≥2 or ≤ −2; *p* < 0.05). **j** GSEA of the MAPK signaling pathway. **k** Western blot showing p-JNK/JNK, p-ERK/ERK, and p-p38/p38 levels in osteoclast precursors following indicated treatments for 48 h with 30 ng/ml M-CSF and 50 ng/ml RANKL. **l** Quantitative analysis of western blots in (**k**). Data are presented as the mean ± SEM (n ≥ 3) (*p < 0.05; **p < 0.01; ***p < 0.001)

As a deacetylase in mitochondria, SIRT3 has been linked to mitochondrial metabolism synthesis and mobility.^28^ The protective role of SIRT3 in many diseases through the scavenging effect on ROS has been demonstrated in multiple studies.^29–31^ Therefore, we subsequently investigated whether the miR-6359-mediated SIRT3 silencing could alter ROS levels in osteoclast precursors. We used SIRT3 siRNA to lower or silence SIRT3 expression to simulate the miR-6359-induced SIRT3 inhibition effect. According to the western blot and qRT-PCR analysis results, siRNAs, especially siRNA3, effectively knocked down SIRT3 expression (Fig. 4a, b). Following that, we used oxidation-sensitive dyes, DCFH-DA and MitoSOX, to visualize the oxidative fluorescent signals with a fluorescence microscope to examine the role of intracellular ROS in osteoclast precursors with SIRT3 siRNAs or vehicle infection. Compared to the control group, ROS-positive cells, marked by DCF-DA and MitoSOX staining after siRNA treatment, were significantly increased, indicating SIRT3 inhibition could promote ROS production in both the mitochondria and cytosol under osteoclast differentiation (Fig. 4c–f). Furthermore, TRAP staining revealed that SIRT3 knockdown increased osteoclastic formation (Fig. 4g, h), indicating SIRT3 involvement in osteoclastogenesis.

The kyoto encyclopedia of genes and genomes (KEGG) pathway and molecular function enrichment analysis of differential genes revealed that the MAPK signaling pathway was significantly enriched (Fig. 4i). We then performed the gene set enrichment analysis (GSEA) of the MAPK signaling pathway, which indicated that the MAPK gene set was over-represented in osteoclast precursors following VPA treatment (Fig. 4j). The involvement of the MAPK signaling pathway in osteoclast formation is well demonstrated in previous research.^32,33^ As a result, we investigated whether SIRT3 siRNA activated the signaling cascades during osteoclast differentiation. As examined by western blot analysis, RANKL supplementation significantly increased the phosphorylation of p38 MAPK, extracellular signal-regulated protein kinase (ERK), and JNK. Additionally, the p38 MAPK, ERK, and JNK phosphorylation was further intensified by SIRT3 siRNA3 exposure. However, the phosphorylation of the three MAPKs was evidently inhibited when MitoQ (a ROS scavenger) was added (Fig. 4k, l), showing that SIRT3 knockdown could effectively activate the MAPK signaling pathway, with the effect, at least partially, being attributed to the SIRT-mediated ROS generation. Taken together, these findings reveal that VPA treatment increases osteoclastogenesis through the miR-6359/SIRT3/MAPK axis in osteoclast precursors.

### Construction of E-sEVs

**Fig. 5:**
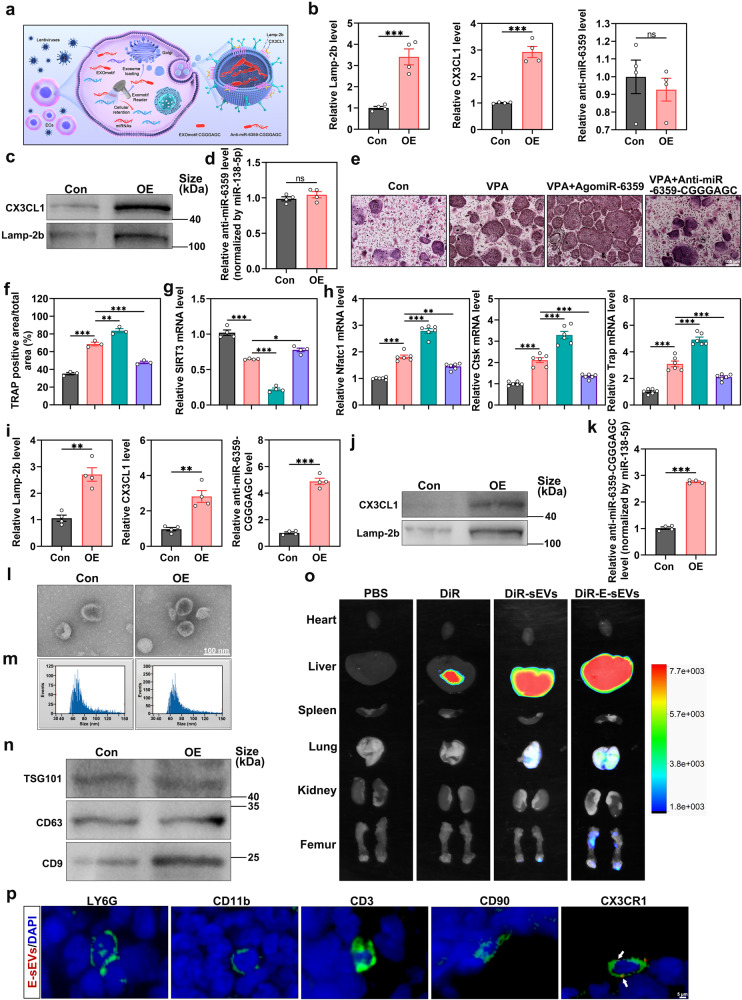
Construction of E-sEVs. **a** Schematic diagram showing the construction process of E-sEVs. **b** qRT-PCR analysis of Lamp-2b and CX3CL1 as well as anti-miR-6359 expression levels in osteoclast precursors infected with the Lamp-2b and CX3CL1-overexpressed lentivirus or negative controls (*n* = 4). **c** Western blot analysis of the expression of Lamp-2b and CX3CL1 proteins in sEVs derived from ECs after infection. **d** qRT-PCR analysis of anti-miR-6359 in sEVs (*n* = 4). **e**, **f** TRAP staining of osteoclasts (**e**), and their corresponding quantifications (**f**) (*n* = 3). Scar bar: 100 μm. **g**, **h** qRT-PCR detection of SIRT3 (**g**), Nfatc1, Ctsk and Trap (**h**) mRNA levels in osteoclast precursors following corresponding treatments (*n* = 4 or 6). **i** qRT-PCR analysis of Lamp-2b and CX3CL1 as well as anti-miR-6359-CGGGAGC expression levels in osteoclast precursors infected with pLenti-U6-anti-miR-6359-CGGGAGC-EF1N promoter-Lamp-2b (CX3CL1-Extra-sv40-puro)-sv40-puro lentivirus or negative controls (*n* = 4). **j** Western blot analysis showing the Lamp-2b and CX3CL1 protein levels in sEVs. **k** qRT-PCR analysis of anti-miR-6359-CGGGAGC in sEVs (*n* = 4). **l** SEVs morphology. Scar bar: 100 nm. **m** SEVs diameter distribution. **n** SEVs marker analysis by western blot. (o) Organ fluorescence of mice injected intravenously with PBS, dissolved DiR equivalents, DiR-labeled sEVs and DiR-labeled E-sEVs. **p** The femurs from mice treated with DiR-labeled E-sEVs were harvested and subjected to immunofluorescence staining for LY6G, CD11b, CD3, CD90 and CX3CR1. Scar bar: 5 μm. Data are presented as the mean ± SEM (*n* ≥ 3) (*p < 0.05; **p < 0.01; ***p < 0.001)

The above findings indicate that the promotive effect of VPA on osteoclast differentiation could be attributed to the VPA-mediated miR-6359 upregulation. In this regard, to prevent the VPA-induced bone loss, we subsequently opted to reverse the effect of VPA-enhanced osteoclastogenesis using anti-miR-6359 given that anti-miRs provided higher stability and effective inhibition of specific miRNA.^34^ We established engineered sEVs targeting osteoclast precursors by introducing a specific osteoclast precursor receptor or ligand into the sEVs surface. This approach ensures an adequate anti-miR-6359 concentration for delivering to osteoclast precursors in bone tissue based largely on the fact that sEVs are administering therapeutically relevant compounds. Herein, CX3CR1, a marker for osteoclast precursors, was important for the process of cells expressing the CX3CR1 ligand (CX3CL1) recruitment.^23,35^ The endothelial cells (ECs) were transfected with the lentivirus encoding the extracellular end of CX3CL1 fused with the N-terminal of Lamp-2b, which is present on the sEVs membrane, to produce sEVs with high CX3CL1 expression on their membranes (Fig. 5a). The effective overexpression of CX3CL1 and Lamp-2b in ECs was validated by qRT-PCR analysis (Fig. 5b). Subsequently, sEVs were then harvested from the supernatant of lentivirus-transfected cultured ECs. Additionally, we examined the presence of CX3CL1 and Lamp-2b proteins, as well as anti-miR-6359 in the sEVs though western blot and qRT-PCR analysis, respectively. The findings revealed that CX3CL1 and Lamp-2b expression was significantly increased as compared to sEVs isolated from cells transfected with the control lentivirus (Fig. 5c). However, the contents of anti-miR-6359 level in the ECs and sEVs were very low and did not increase following transfection with the lentivirus (Fig. 5b, d).

Exogenous RNAs can be directly loaded into sEVs by electroporation,^36,37^ lipofection,^38^ sonication,^39^ and calcium chloride.^40^ However, several studies revealed that RNAs loaded directly into sEVs might not be functionally active when taken up by recipient cells,^41,42^ potentially leading to the destruction of the membranous integrity of sEVs to some extent. Therefore, cells that actively and specifically sort miRNAs into sEVs might be a better choice for producing anti-miR-6359-carrying sEVs. In this regard, we introduced an EXOmotif of ECs (CGGGAGC) into the 3’ half of the anti-miR-6359 (anti-miR-6359-CGGGAGC) and covered that anti-miR-6359-CGGGAGC treatment could block the increase of VPA-induced osteoclastogenesis and related genes expression, as well as recover the VPA-induced SIRT3 downregulation, as with antagomiR-6359 effects in vitro (Fig. 5e–h).^43^ Therefore, the ECs were transfected with the lentivirus (pLenti-U6-anti-miR-6359-CGGGAGC-EF1N promoter-Lamp-2b (CX3CL1-Extra- sv40-puro)-sv40-puro lentiviruses) to generate engineered sEVs highly expressing CX3CL1 and naturally and specifically carrying anti-miR-6359-CGGGAGC (Fig. 5a). We then collected the transfected ECs-derived sEVs and discovered that Lamp-2b, CX3CL1 and anti-miR-6359-CGGGAGC were highly expressed in both ECs and ECs-derived sEVs compared to the negative control group (Fig. 5i–k). Furthermore, the sEVs were identified and characterized. Specifically, the transmission electron microscope (TEM) revealed that sEVs had the shape of cups (Fig. 5l), and nanoparticle tracking analysis (NTA) indicated that the sEVs showed a mean diameter of (77.63) nm and (77.15) nm, respectively (Fig. 5m). The sEVs protein markers, including TSG101, CD63, and CD9, were examined through western blot analysis (Fig. 5n), which showed the successful generation of the CX3CL1^+^ anti-miR-6359-CGGGAGC^+^ sEVs (E-sEVs) though genetically engineering strategy. Furthermore, TRAP staining analysis revealed that E-sEVs exhibited suppressive capacities of VPA-induced osteoclast differentiation in vitro (Supplementary Fig. 4a, b).

The bone-targeting properties of the E-sEVs were subsequently assessed. The Bruker MI SE system detection results showed that DiR-E-sEVs specifically targeted bone tissue and were more concentrated in the liver, lung and bone compared to PBS, DiR and DiR-sEVs groups (Fig. 5o). Following that, femurs were extracted from DiR-E-sEVs treated mice, and immunofluorescence for LY6G, CD11b, CD3, CD90 and CX3CR1 in femur sections was performed. The co-localization between E-sEVs and CX3CR1 was observed (white arrows), but there was no or little evidence of co-locazilzation between E-sEVs and LY6G, CD11b, CD3 and CD90 (Fig. 5p). Meanwhile, Hematoxylin and Eosin (H&E) staining of the heart, liver, spleen, lung and kidneys revealed no E-sEVs treatment-induced toxicity (Supplementary Fig. 4c). To the best of our knowledge, this is the first study to produce genetically engineered osteoclast precursor-targeted sEVs, potentially offering guidance for sEVs delivery systems for osteoclast precursors in osseous tissue.

### E-sEVs prevent VPA-induced bone loss

**Fig. 6:**
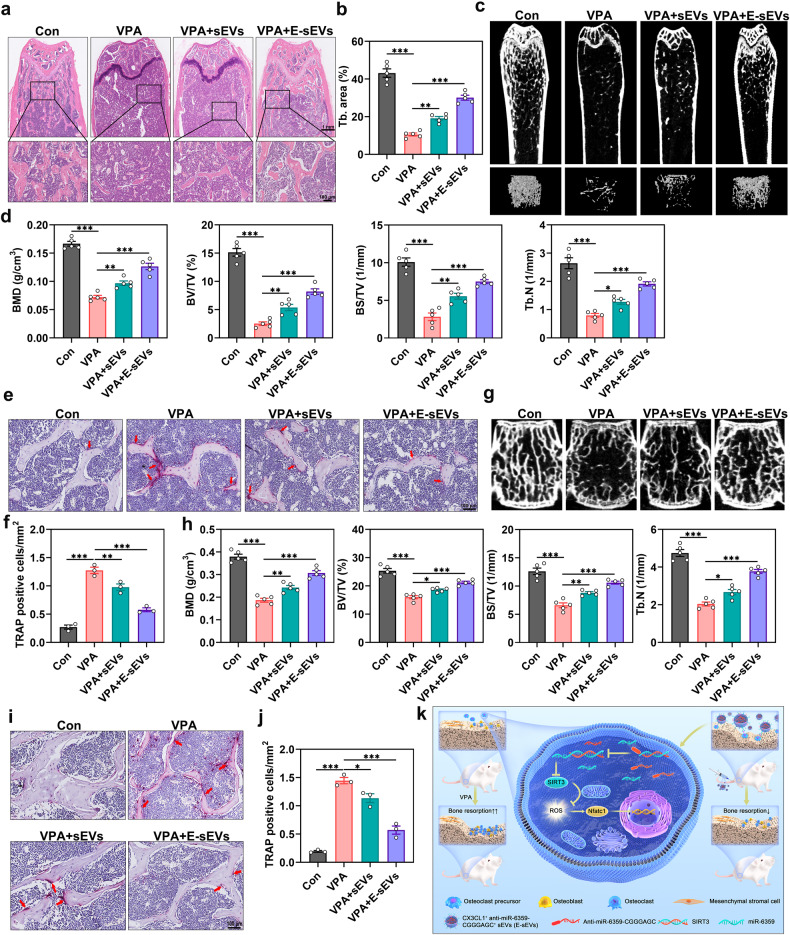
E-sEVs prevent VPA-induced bone loss. Mice aged eight weeks were treated with vehicle or VPA in combination with sEVs (VPA+sEVs) or E-sEVs (VPA + E-sEVs) for four weeks. **a** H&E staining of distal femur sections (*n* = 5). Scar bar: 1 mm and 100 μm. **b** Quantitative assessment of Tb.N in (**a**). **c** Micro-CT images of the femurs from control, VPA, VPA+sEVs and VPA + E-sEVs groups (*n* = 5). **d** Morphometric analyses of trabecular bone. **e**, **f** TRAP staining of the femurs isolated from mice subjected to various treatments and the corresponding quantification (*n* = 3). Scar bar: 100 μm. **g** Micro-CT images in the lumbar spine after corresponding treatments (*n* = 5). **h** Corresponding morphometric measurements of trabecular bone in (**g**). **i**, **j** TRAP staining of the lumbar spine sections and quantification of TRAP-positive cells (*n* = 3). Scar bar: 100 μm. **k** Schematic diagram showing the proposed mechanism of VPA-induced bone loss and how E-sEVs prevent it. Data are presented as the mean ± SEM (n ≥ 3) (*p < 0.05; **p < 0.01; ***p < 0.001)

We examined the effect of E-sEVs on VPA-induced bone loss in an in vivo mouse model to further investigate their efficacy. As shown by H&E staining, the low-bone mass phenotype was observed after VPA treatment, and sEVs treatment appeared to slightly prevent VPA-induced bone loss, which is consistent with a previous study.^44^ On the other hand, E-sEVs significantly prevented VPA-induced bone loss (Fig. 6a, b). The micro-CT analysis of the femurs revealed that E-sEVs treatment significantly reversed VPA-induced low bone mass and deterioration of the trabecular microarchitecture (Fig. 6c). Morphometric analysis of trabecular parameters, such as BMD, BV/TV, BS/TV, and Tb.N, further confirmed this finding (Fig. 6d). The lumbar spine exhibited a similar pattern of bone loss, with a substantial reduction in BMD, BV/TV, BS/TV, and Tb.N upon VPA stimulation. However, these effects were reversed by E-sEVs treatment (Fig. 6g, h). Furthermore, the TRAP staining of femurs and lumbar spine revealed that E-sEVs treatment could dramatically alleviate the VPA-induced increase in osteoclast number compared to the sEVs and VPA groups (Fig. 6e, f, i, j).

Bisphosphonates which exert a robust effect on various kinds of osteoporosis, are commonly used for clinical osteoporosis treatment. In this regard, we subsequently compared medication effects on VPA-induced osteoporosis. We discovered that although E-sEVs exhibited weaker therapeutic effects compared to the bisphosphonate treatment doses during VPA administration over a four-week period, they had some positive effects (Supplementary Fig. 5a–c). Interestingly, the effect of E-sEVs on VPA-induced bone loss was preferential to the bisphosphonate treatment effect in eight weeks of VPA treatment (Supplementary Fig. 5d–f), implying that the therapy is more beneficial in the long term. Additionally, we tested the effect of E- sEVs in ovariectomy (OVX) and glucocorticoid-induced osteoporotic mice models. As show in supplementary Fig. 6a–c, sEVs treatment alleviated the OVX-induced bone loss, but no difference was observed between sEVs group and E-sEVs group, implying that the beneficial effect could be attributed to ECs-derived sEVs rather than E-sEVs, in accordance with previous research.^44^ Moreover, the sEVs group in glucocorticoid (dexamethasone)-induced osteoporotic model exhibited a similar pattern (supplementary Fig. 6d–f). Overall, these findings suggest that implementing E-sEVs targeting osteoclast precursors and carrying anti-miR-6359-CGGGAGC might be more effective treatment for preventing VPA-induced bone loss than other osteoporotic animal models.

## Discussion

This study aimed to investigate the impact of VPA on bone health and develop strategies for counteracting its negative effects on osseous tissue. First, we confirmed that patients and mice undergoing VPA treatment had accelerated bone turnover and bone mass loss in the femurs and lumbar spine. Subsequently, the adverse effects on bone density and bone mass were proved to be related to the enhanced osteoclast formation and activity induced by VPA. This phenomenon could be attributed to a dramatic increase in miR-6359 levels following treatment with VPA, which downregulated SIRT3 expression in osteoclast precursors and subsequently stimulated an elevation of the intracellular ROS levels, resulting in the activation of the MAPK signaling pathway and osteoclastogenesis, thereby accelerating bone loss. To counteract the bone loss caused by VPA, we developed the E-sEVs that specifically target osteoclast precursors in bone tissue and naturally carry anti-miR-6359-CGGGAGC in accordance with the underlying mechanism of VPA-induced bone loss. The experiments revealed that E-sEVs exhibited decent osteoclast precursor targeting and effectively reduced VPA-induced bone loss in vivo, indicating that they could be a promising nanotherapeutic avenue for treating VPA-induced BMD and bone mass reductions (Fig. 6k).

The effect of VPA on bone health is still being debated and the available evidence is primarily from small studies and case reports on VPA use in individuals with seizure disorders. A previous study revealed that 68% of adult patients undergoing VPA treatment exhibited osteoporosis or osteopenia compared to 22% of the control group and there is a time-effect relationship between VPA use and the progression of osteoporosis.^45^ Multiple studies have elaborated the close link between VPA use and bone loss.^25,46,47^ Contrastingly, other studies found no evidence of osteopenia or abnormal bone metabolism markers in adult patients undergoing VPA treatment.^13,48^ Additionally, clinical studies on VPA use in children appeared to provide conflicting results,^14,25,49^ implying that the relationship between VPA use and bone health is controversial in both children and adults. Furthermore, there are no clinical trials on the effect of VPA on bone health in older patients, and only one study found that intermittent VPA treatment positively influenced bone health in ovariectomized rats.^50^ Herein, we primarily focused on the correlation between VPA use and bone health among adult patients and mice and discovered that VPA treatment was associated with lower BMD and elevated serum levels of bone turnover markers in both patients and mice. Consistent with previous research,^51^ subsequent in vitro experiments confirmed that VPA treatment could promote osteoclast formation and function. However, VPA had a negligible impact on osteoclastic differentiation and formation in vitro. On the other hand, other studies revealed that VPA treatment could also promote bone formation and reduce bone resorption,^52,53^ potentially due to higher VPA doses. Specifically, while higher VPA concentrations may promote osteoclast apoptosis or death, osteogenic lineages may generally tolerate these doses well. Consequently, additional research is required to better understand the effects of higher VPA concentrations on osteoclast and osteoblast activity. Based on these findings, we hypothesized that VPA-induced bone loss was primarily the result of enhanced osteoclast differentiation and activity.

According to previous research,^54^ bone breakdown occurs when osteoclastic resorption exceeds osteoblastic bone formation. Excessive osteoclast activity could cause imbalanced bone homeostasis, resulting in metabolic skeletal diseases, such as metastatic cancers, rheumatic arthritis, and osteoporosis.^55,56^ Research has shown that osteocyte death caused by the stimulation of macrophage-inducible C-type lectins can stimulate osteoclast formation and aggravate bone loss.^57^ Additionally, Vav3,^58^ semaphorin 3 A (SEMA3A),^59,60^ and protein kinase C beta II (PKCβII)^61^ were demonstrated to regulate osteoclast formation and bone loss. Some miRNAs, such as miR-214, were recently reported to also influence osteoclastogenesis and regulate bone mass.^62^ We employed RNA-sequencing to detect the changes in mRNAs or miRNAs in osteoclast precursors to investigate the underlying mechanisms of VPA during osteoclastogenesis. Compared to the vehicle-treated osteoclast precursors, the expression of precursor miR-6359 was increased in VPA-treated osteoclast precursors. We subsequently found that miR-6359 was abundant in osteoclast precursors following VPA stimulation both in vivo and in vitro. Furthermore, the in vivo results showed that blocking miR-6359 with anti-miR-6359-CGGGAGC reduced VPA’s ability to promote osteoclast formation and activity. Combined with the in vitro experiment, these results revealed that miR-6359 was critically involved in VPA-induced osteoclast formation. Given that miR-6359 inhibition did not completely eliminate the positive effects of VPA on osteoclast formation in vitro, other miRNAs or genes may also partially contribute to VPA’s ability to promote osteoclast formation, which needs further elucidation. Interestingly, the VPA-induced miR-6359 upregulation was not observed in other tissues, such as heart, liver, spleen, lung and kidney, and in other cells in bone, including the neutrophils, T lymphocytes, monocytes and BMSCs. Therefore, whether VPA acts via other signaling molecules in various tissue and cells needs to be further investigated. Next, three miRNA databases and OP-related genes were analyzed to identify the common target genes of miR-6359, which revealed that SIRT3 may be the most important target gene directly regulated by miR-6359, as confirmed by western blot and qRT-PCR analysis. Furthermore, studies have shown that SIRT3 protein is associated with osteoclast differentiation and activity. Therefore, Ling W et al. reported that the knockdown of SIRT3 inhibited osteoclastogenesis and alleviated aging and estrogen deficiency-induced bone loss, but it had no effect on the skeleton of young mice.^63^ However, Li N et al. showed that SIRT3 silencing protected young mice from titanium particle-induced bone loss.^64^ Elsewhere, it was found that silencing of SIRT3 increased the ROS level, leading to osteoclastogenesis and bone loss.^27^ Moreover, other scholars have demonstrated that SIRT3 silencing impaired osteoblast differentiation and bone formation and increased bone loss in aged mice.^65–67^ Therefore, these findings suggest that SIRT3 may have divergent effects during osteoclastogenesis and osteogenesis,^68^ and further research is needed to clarity its role. In our study, we found that reducing SIRT3 expression increased ROS levels in osteoclast precursors and osteoclast formation.

To rescue VPA-induced bone loss, we prepared to establish E-sEVs targeting osteoclast precursors and carrying anti-miR-6359. The presence of EXOmotif (CGGGAGC) in ECs facilitated the natural sorting of specific miRNA into sEVs,^43^ and we assumed that E-sEVs isolated from ECs transfected with the lentiviruses had a high bone-targeting ability and were naturally enriched with anti-miR-6359-CGGGAGC, as confirmed by live-animal fluorescence imaging and qRT-PCR analysis, respectively. Subsequently, we employed E-sEVs to treat the VPA-induced bone impairment. H&E and TRAP staining and micro-CT imaging showed that E-sEVs treatment dramatically prevented bone loss in the context of VPA-induced osteoporosis, but no difference was observed between sEVs group and E- sEVs group in OVX and glucocorticoid-induced osteoporotic mouse models, suggesting that E-sEVs might be a specific drug for VPA-induced bone loss with less side effects. Previously, Zhang et al. designed a nanoparticles delivery system targeting different stages of osteoclasts, and confirmed that the system could effectively target the corresponding osteoclasts.^69^ However, our delivery system may be inferior to other engineering sEVs system in terms of biocompatibility and the loading efficiencies of specific miRNA, as reported previously.^70^ Therefore, the system exhibited the following advantages: (1) it is the first system to target osteoclast precursors to reduce VPA-associated side-effects on bone. (2) The introduction of CGGGAGC into anti-miR-6359 greatly increased their concentration in the E-sEVs, which protected the membranous integrity of the sEVs compared with other methods such as electroporation, lipofection, sonication, and calcium chloride. (3) The E-sEVs carrying anti-miR-6359-CGGGAGC were highly efficient.

In summary, this study demonstrates that VPA treatment enhances osteoclast formation and activity via the miR-6359/SIRT3/MAPK signaling pathway in vivo and in vitro, leading to bone loss. This is the first study demonstrated that VPA exacerbates bone loss in vivo through miR-6359. Moreover, the results indicate that the E-sEVs could target osteoclast precursors in bone and significantly reverse VPA-induced bone loss in vivo. Therefore, the E-sEVs are potential therapeutic agents for treatment of VPA-related bone loss.

## Materials and methods

### Reagents

VPA was obtained from MedChemExpress (New Jersey, USA). GAPDH, JNK, p-JNK, p38, p-p38, ERK, p-ERK, SIRT3, CD3, LY6G and CX3CR1 antibodies were purchased from ABclonal (Wuhan, China), and antibodies against CX3CL1 and Lamp-2b were bought from Abcam. Osteocalcin antibody was purchased from Takara (Japan, M173). Penicillin-streptomycin was purchased from Solarbio (Beijing, China). The RANKL and M-CSF were bought from R&D Systems (Minnesota, USA). The cell culture plates were from NEST (Jiangsu, China). Minimum Essential Medium Alpha (α-MEM) and fetal bovine serum (FBS) were purchased from Gibco (Grand Island, NY, USA). CCK-8 (Kumamoto, Japan) was bought from Dojindo. The enzyme-linked immunosorbent assay (ELISA) kits were purchased from CUSABIO and Elabscience (Wuhan, China). TRAP staining kit was obtained from Sigma–Aldrich (St. Louis, MO, USA).

### Samples of human peripheral blood

This study was conducted in accordance with the Helsinki Declaration, and it was approved by the institutional review board at Union Hospital, Tongji Medical College, Huazhong University of Science and Technology. All subjected provided informed consent to participate in the study before sample collection. The sample information is provided in Supplementary Tables 1, 2.

### Animal treatment

All animal experiments were performed in line with the procedures and guidelines of ARRIVE of Laboratory Animal Center of Tongji Medical College, Huazhong University of Science and Technology. The mice were kept in the animal facility with a twelve-hour day and night cycle, temperature (22°C) and humidity (60%). Mice C57BL/6 aged 8 weeks were randomly assigned to either the control group (treated with vehicle) or the treatment group (treated with VPA or VPA+sEVs or VPA + E-sEVs). The mice were injected via the tail vein thrice a week with sEVs (30 μg/100 μl) or an equal volume of solvent (PBS; 100 μl), along with VPA (200 mg/kg) or vehicle (saline) (intraperitoneally, thrice a week) for four weeks. At the end of the treatment period, the mice were sacrificed in a humane manner, and femurs and lumbar spine were collected for micro-CT scanning. Next, the blood samples were harvested and the supernatant was preserved in the freezer at –80 °C after centrifuging for five minutes at 1000×g.

### Quantitative real-time PCR (qRT-PCR)

Total RNA was extracted using Trizol (Invitrogen) following the manufacturer’s protocols. The purity and concentration of the RNA was determined by measuring the optical density at 260 nm/280 nm. 1000 ng of total RNA was used to synthesize cDNA. qRT-PCR was performed on equal amounts of cDNA using 2×AceQ qPCR SYBR Green Master Mix (YEASEN, China). The GAPDH gene served as the internal control. The mRNA level of the target gene was determined using the 2^ΔΔCt^ method.

The primer sequences used for qRT-PCR are shown in Supplementary Table 3.

### RNA sequencing (RNA-seq)

The total RNA was obtained as described in qRT-PCR methods. Briefly, following RNA extraction, the sequencing library was constructed according to the manufacturer’s protocol using RNA Library Prep Kit (Vazyme Biotech, Nanjing, China). The libraries were quantified with a Qubit and sequenced on an Ilumina Novaseq 6000 platform. Low-quality reads were eliminated using Skewer (V0.2.2) to obtain clean reads, and FastQC was used to conduct quality control analyses (V0.11.5). DESeq2 was employed to perform differential expression analysis (V1.16.1). A *p* value < 0.05, a foldchange ≥ 2 or a foldchange ≤ -2 was set as the cutoff for differentially expressed genes (DEGs).

### Preparation and culture of osteoclasts

Osteoclast precursors were prepared as previously described.^71^ In brief, cells were extracted from the femurs and tibias of five-week-old mice. The samples were treated with blood cells (RBC) lysis buffer (Servicebio) to eliminate RBC, and the cells were cultured in medium overnight. Nonadherent cells were transformed into osteoclast precursors through incubation with 30 ng/ml M-CSF for five days. Subsequently, the specified quantity of osteoclast precursors was seeded in plates for subsequent experiments.

### Osteoclastogenesis assay in vitro

Approximately 1 × 10^4^ osteoclast precursors were seeded into each well of a 96-well plate and cultured overnight. To induce osteoclast differentiation, a mixture of osteoclastic induction medium with indicated treatments was added. The TRAP staining assay was performed following the manufacturer’s instructions five days later. Osteoclasts were defined as cells with more than three nuclei that were positive for TRAP staining. The osteoclastogenic ability was calculated as the TRAP-positive area versus total area.

### Western blot analysis

Approximately 4 × 10^5^ osteoclast precursors were seeded in six-well plates and cultured overnight. The cells were treated with RIPA lysis buffer containing a proteinase inhibitor cocktail and PMSF to extract the total protein following various treatment. 10 µg of protein was separated using a 10% SDS-PAGE gel, and were electrophoretically transferred to polyvinylidene fluoride (PVDF) membranes. The membrane was blocked in 5% nonfat milk for one hour, and then incubated with the corresponding antibodies overnight at 4 °C. After ten-minute TBS-T washes, the membranes were incubated with secondary antibodies for one hour at room temperature. They were kept in chemiluminescent substrate and visualized with the Bio-Rad Image Capture System after PVDF membranes were washed three times with TBST.

### Immunofluorescence staining

Approximately 5 × 10^4^ osteoclast precursors were seeded into each well of a 12-well plate and cultured overnight. The effects of VPA on cells transfected with agomiRs or antagomiRs were examined in the presence of 30 ng/ml M-CSF and 50 ng/ml RANKL. The cells were then fixed in 4% paraformaldehyde (PFA) for 20 min, permeabilized with 0.5% Triton X-100 for 10 min, and then blocked with 5% goat serum for 30 min. They were washed three times with PBS, incubated with primary antibodies (SIRT3; diluted 1:100) at 4 °C overnight, and then stained for 5 min with 4,6-diamidino-2-phenylindole (DAPI, Solarbio).

### miR-6359 fluorescent in situ hybridization (miR-6359-FISH)

miR-6359-FISH was used to explore the location of miR-6359 by a Fluorescent in Situ Hybridization Kit (Genepharma; shanghai, China) following the manufacturer’s instructions.

### ELISA assay

Blood supernatants from both mice and patients were collected, and the serum concentrations of TRACP-5b, β-CTX, PINP, and BALP were subsequently assessed using ELISA kits (Cusabio and Ebabscience; Wuhan, China) following the provided instructions.

### SIRT3 siRNA transfection

Osteoclast precursors were transfected with either SIRT3 small interfering RNA (siRNA) or nontarget control siRNA (GenePharma; Shanghai, China) for 24 hours using Lipofectamine^TM^ 3000 Transfection Reagent. The sequences of SIRT3 siRNA were as follows: SIRT3 siRNA1: 5’-GACUUCGCUUUGGCAGAUC (dTdT)-3’ (sense) and 5’- GAUCUGCCAAAGCGAAGUC (dTdT)-3’ (antisense); SIRT3 siRNA2 5’-CACUAGGGCAAUCUAGCAU (dTdT)-3’ (sense), 5’-AUGCUAGAUUGCCCUAGUG (dTdT)-3’ (antisense); SIRT3 siRNA3 5’-CUGCCUCAAAGCUGGUUGA (dTdT)-3’ (sense) and 5’- UCAACCAGCUUUGAGGCAG (dTdT)-3’ (antisense).

### Micro-CT analysis

The femurs and lumbar spine were subjected to analysis using micro-CT (Bruker SkyScan 1176 scanner mCT system). Scanning was performed with parameters set at 37 kV and 121 mA, resulting in 300 section planes. BMD, BV/TV, BS/TV, and Tb. N were acquired through NRecon and CTan software.

### H&E and TRAP staining

The femurs and lumbar spine were collected from mice, and then fixed in 4% PFA. The samples were decalcified using 10% tetracycline-EDTA (Servicebio) for two weeks. H&E and TRAP staining were performed on 4-μm-thick paraffin-embedded sections following the manufacturer’s instructions (Sigma-Aldrich). Histological images were collected using a microscope and analyzed using ImageJ software.

### Detection of Intracellular ROS

To assess the intracellular ROS levels, a fluorescent dye, 10 mM DCF-DA (Beyotime; Shanghai, China), was employed. Cells were transfected with SIRT3 siRNA for 24 hours, followed by staining with DCF-DA for 20 min at 37 °C after three washes. ROS-induced green fluorescence of DCF-DA was recorded using 488 nm laser excitation.

The levels of ROS in the mitochondria were detected using the MitoSOX Red mitochondrial superoxide indicator (Thermo Fisher Scientific). After SIRT3 siRNA transfection, cells were loaded with 5 μM MitoSOX at 37 °C for 10 min. Mitochondrial ROS-induced red fluorescence of MitoSOX was measured using 580 nm laser excitation.

### Construction of stable CX3CL1 and anti-miR-6359-CGGGAGC-overexpressing lentiviruses and transductions

pLenti-U6-anti-miR-6359-CGGGAGC-EF1N promoter-Lamp-2b (CX3CL1-Extra- sv40-puro)-sv40-puro lentiviruses and control lentiviruses were bought from GeneChem (Shanghai, China). They were used to transfect SVEC (mouse lymphatic endothelial cells, ECs). Briefly, the lentiviruses were seeded in SVEC with a multiplicity of infection (MOI) of 20, and the corresponding empty vectors were used as controls. Cells overexpressing CX3CL1 and anti-miR-6359-CGGGAGC were harvested following 4~6 days of puromycin (Sigma-Aldrich) selection at a final concentration of 2 μg/ml.

### Characterization of sEVs

SEVs were isolated as previously described.^72^ Briefly, cells were seeded into T75 culture flasks and cultured until they reached 70~80% confluency. Next, the cells were incubated in the sEVs-free medium. After 24 hours, the cell supernatant was harvested and centrifuged at 300 × *g* for 10 min to eliminate debris and unattached cells. The supernatant was then centrifuged twice, first for 40 min at 10,000 × *g* and then for 70 min at 120,000 × *g*. Precipitates were dissolved in 30 ml of PBS, and the sample was centrifuged again at 120,000 × *g* for 70 min. Finally, the supernatant was discarded and the precipitates were resuspended in 200 μl of PBS before they were frozen at –80 °C. The BCA protein assay kit from Beyotime (Shanghai, China) was used to measure the concentration of sEVs. The sEVs were identified by examining their morphology and particle size distribution using TEM and NTA. The western blot analysis was used to quantify specific marker proteins in sEVs, including TSG101, CD63 and CD9.

### Dual-luciferase reporter assay

The direct interaction between miR-6359 and SIRT3 was validated through the dual luciferase reporter assay. Osteoclast precursors were transfected with recombinant plasmids pmirGLO-SIRT3 and pmirGLO-SIRT3-mut (GenePharma), in conjunction with agomiR-6359 and agomiR-NC, for a duration of 48 h. Subsequently, luciferase activity was quantified using the Dual-Luciferase Reporter Assay System.

### SEVs labeling and trace

SEVs (1 μg/μl) were labeled with DiR. For in vivo fluorescence tracing of sEVs, mice were injected with 100 μg of DiR-labeled sEVs via the tail vein. The localization of sEVs in various organs was examined using the Bruker MI SE system after an eight-hour duration.

### Antibody staining and flow cytometry analysis

Following VPA treatment in mice, cell suspensions from the femurs and tibias were collected and washed twice with PBS. The supernatant was removed, and the cells were resuspended in 100 μl PBS containing the fluorochrome-coupled antibodies against the indicated antigens. They were incubated for 30~40 min at 4 °C in the dark, and washed twice with PBS and resuspended in fresh PBS for flow cytometry. Flow cytometry data were collected using BD FACSDiva Software 8.0.1, and subsequently analyzed with FlowJo software 10.8.1. The antibodies employed for flow cytometry are detailed in Supplementary Table 4.

### Statistical analysis

All analyses were conducted using GraphPad Prism 8.0.2. Normality of data distribution was evaluated using Shapiro-Wilk test. A two-tailed unpaired t-test was utilized to compare the mean value between two conditions or when sample size was too small to test normality of data distribution. For more than two conditions, if normally distributed, one-way analysis of variance was used with Sidak’s multiple comparisons test or Dunnett’s multiple comparisons test. For non-normally distributed data, we employed the nonparametric two-tailed Mann–Whitney test for comparing means between two conditions or the Kruskal–Wallis test with Dunn’s multiple comparisons test for more than two conditions. Each experiment was conducted with a minimum of three replicates, and the results are expressed as the mean with standard error of measurement (SEM). Statistical significance in this study was defined as a *p* value less than 0.05.

### Supplementary information

Supplementary Materials

## Data Availability

The authors will provide the study’s data upon reasonable request. The RNA-seq data have been deposited in Gene Expression Omnibus (GEO) with the following accession numbers: GSE245995 according to the journal publication policy.
