# Supplementary material for: Bone-targeting engineered small extracellular vesicles carrying anti-miR-6359-CGGGAGC prevent valproic acid-induced bone loss
Source: Signal Transduct Target Ther. 2024 Jan 22;9:24. doi: 10.1038/s41392-023-01726-8 (PMC10800355; doi:10.1038/s41392-023-01726-8)
Supplement: Supplementary file 1 — Supplementary Materials [file 41392_2023_1726_MOESM1_ESM.docx]

Supplementary Materials for

Bone-targeting engineered small extracellular vesicles carrying anti-miR-6359-CGGGAGC prevent valproic acid-induced bone loss

Xudong Xie^1,2＃^, Peng Cheng^1,2＃^, Liangcong Hu^1,2＃^, Wu Zhou^1,2^^＃^, Detai Zhang^3＃^, Samuel Knoedler^4,5^, Guodong Liu^6^, Yuan Xiong^1,2^, Hang Xue^1,2^, Yiqiang Hu^1,2^, Barbara Kern^7,8^, Doha Obed^4,9^, Adriana C. Panayi^4,10^, Lang Chen^1,2^, Chenchen Yan^1,2^, Ze Lin^1,2^, Guandong Dai^11^, Bobin Mi^1,2^*, Yingze Zhang^1,12^*, Guohui Liu^1,2^*

Correspondence to: [mibobin@hust.edu.cn](mailto:mibobin@hust.edu.cn); drzhangyz@126.com; liuguohui@hust.edu.cn

**This PDF file includes:**

Supplementary Materials and Methods

Supplementary Fig. 1-6

Supplementary Tables 1-4

Supplementary Materials and Methods

**CCK8**

Approximately 1×10^4^ cells were planted in 96-well plates and cultured overnight. Osteoclast differentiation was induced by adding 30 ng/ml M-CSF and 50 ng/ml RANKL in combination with various concentrations of VPA for five days. The absorbance was measured at 450 nm after two hours of incubation in serum-free medium with CCK8 reagent.

**Pit formation assay**

Bovine bone slices were placed in 96-well plates and seeded with approximately 1×10^4^ osteoclast precursors. After 24 hours, cells were incubated with indicated treatments in the presence of 30 ng/ml M-CSF and 50 ng/ml RANKL. The culture medium was discarded after seven days, and 0.5% sodium hypochlorite was utilized to clean the surface. The bovine bone slices were then washed twice in PBS and allowed to dry for 12 hours in a freeze dryer. The resorbing area was subsequently observed with a scanning electron microscope (SEM) and analyzed with ImageJ software.

**Osteogenesis properties in vitro**

BMSCs were cultured in 12-well plates at a density of 2× 10^5^ cells per well and grown until approximately 70% confluence. The culture medium was replaced by the osteogenic differentiation medium (Cyagen Biosciences) containing 10% (v/v) FBS, 1% (v/v) penicillin–streptomycin, 2 mM L-glutamine, 50 μM ascorbate, 10 mM β-glycerophosphate, and 100 nM dexamethasone. The culture medium was changed every 2~3 days. After 14 days, cells were washed thrice with PBS, and then fixed with 4% PFA for 15 min. Finally, cells were stained with an Alkaline Phosphatase Assay Kit ( Beyotime, Shanghai, China) according to the manufacturer’s instructions.

Alizarin Red S (ARS) staining was performed after 21 days of induction. After fixing with 4% PFA, each well was treated with Alizarin Red S solution (Cyagen Biosciences) and incubated in the dark for 30 min. Then, the images were obtained by microscopy. Finally, quantification of calcium deposition was performed by elution of ARS with 10% (W/V) cetylpyridinium chloride in 10 mM sodium phosphate (PH 7.0) for 1 h at room temperature, and absorbance of the eluted dye was measured at 570 nm.

**Animal treatment**

The Laboratory Animal Center of Tongji Medical College, Huazhong University of Science and Technology, abided by ARRIVE standards and procedures to conduct all animal experiments. The animal facility with a twelve-hour day and night cycle, temperature (22℃) and humidity (60%) were kept.

Ovariectomy (OVX) model and treatment: Eight-week-old female mice were weighed and anesthetized with 1% pentobarbital by intraperitoneal injection, and then subjected to bilateral OVX or a sham operation. Four weeks later, mice were treated with vehicle, small extracellular vesicles

(sEVs) or engineering sEVs (E-sEVs) thrice per week for four weeks. Then the femurs were collected for further analysis.

Glucocorticoid (dexamethasone)-induced osteoporotic mice model and treatment: Eight-week-old C57BL/6 mice were randomly assigned to four groups: control group, treatment with vehicle; Dexamethasone group (Dex), treatment with Dex by intramuscular injection for four weeks; Dex+sEVs group, co-treatment with Dex and sEVs for four weeks; Dex+E-sEVs group, co-treatment with Dex and E-sEVs for four weeks.

**Supplementary Figure
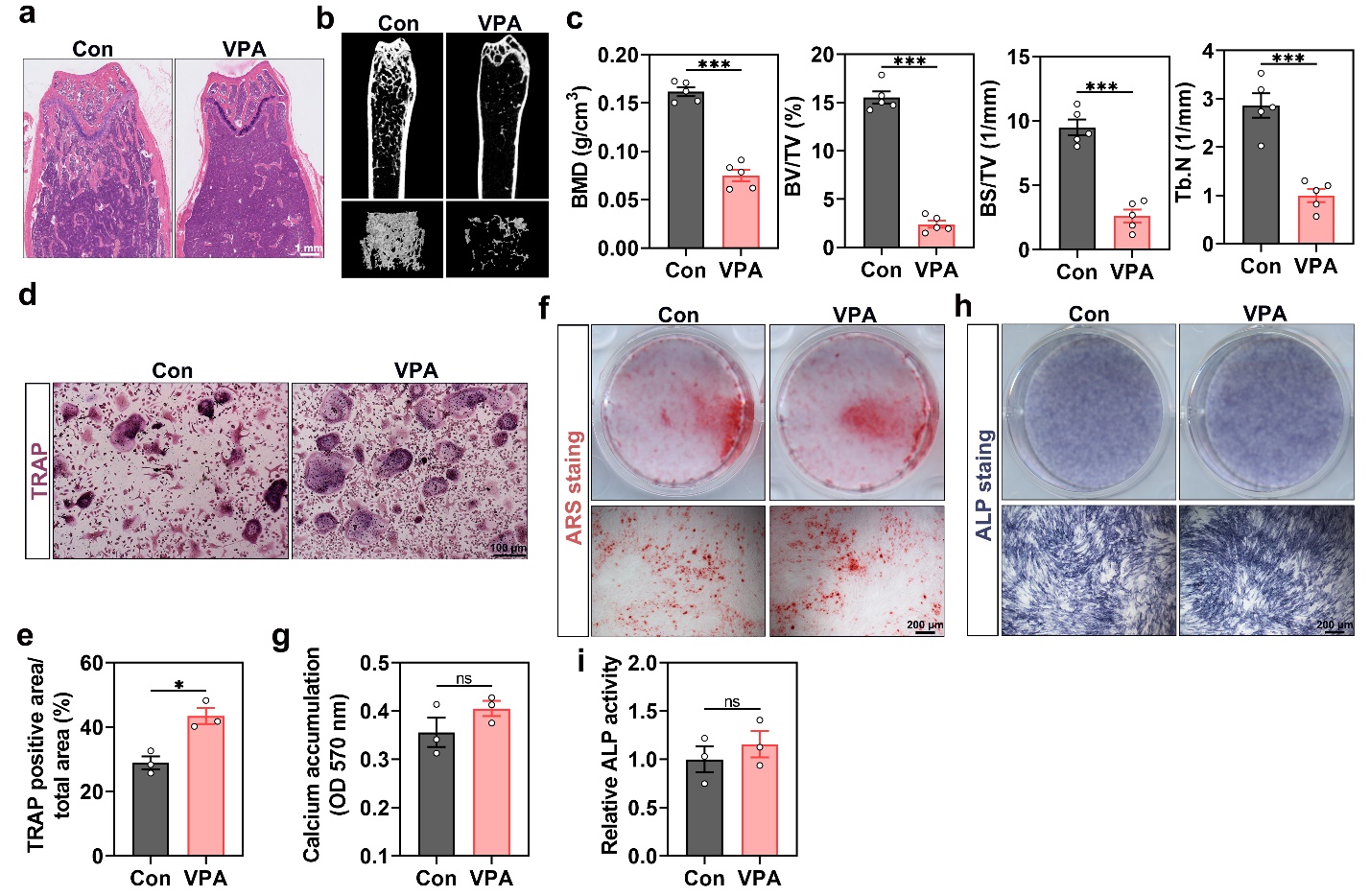
**

**Supplementary Fig 1**: (a) Representative images of H&E staining in femurs (n=5). Scar bar: 1 mm. (b, c) Micro-CT images of the femurs in female mice (b) and quantification analysis (c) (n=5). (d) TRAP-positive multinuclear cells images (n=3). Scar bar: 100 μm. (e) Quantitative analysis of TRAP-positive area/total area (%) (n=3). (f, g) Representative images of ARS staining (f) and quantification of ARS staining (g) (n=3). Scar bar: 200 μm. (h, i) Osteoblastic differentiation of BMSCs was determined by ALP staining (h) and quantitative analysis of ALP activity (i) (n=3). Scar bar: 200 μm. All results are displayed as means ± SEM (n≥3)(**p*<0.05; ***p*<0.01; ****p*<0.001).

**
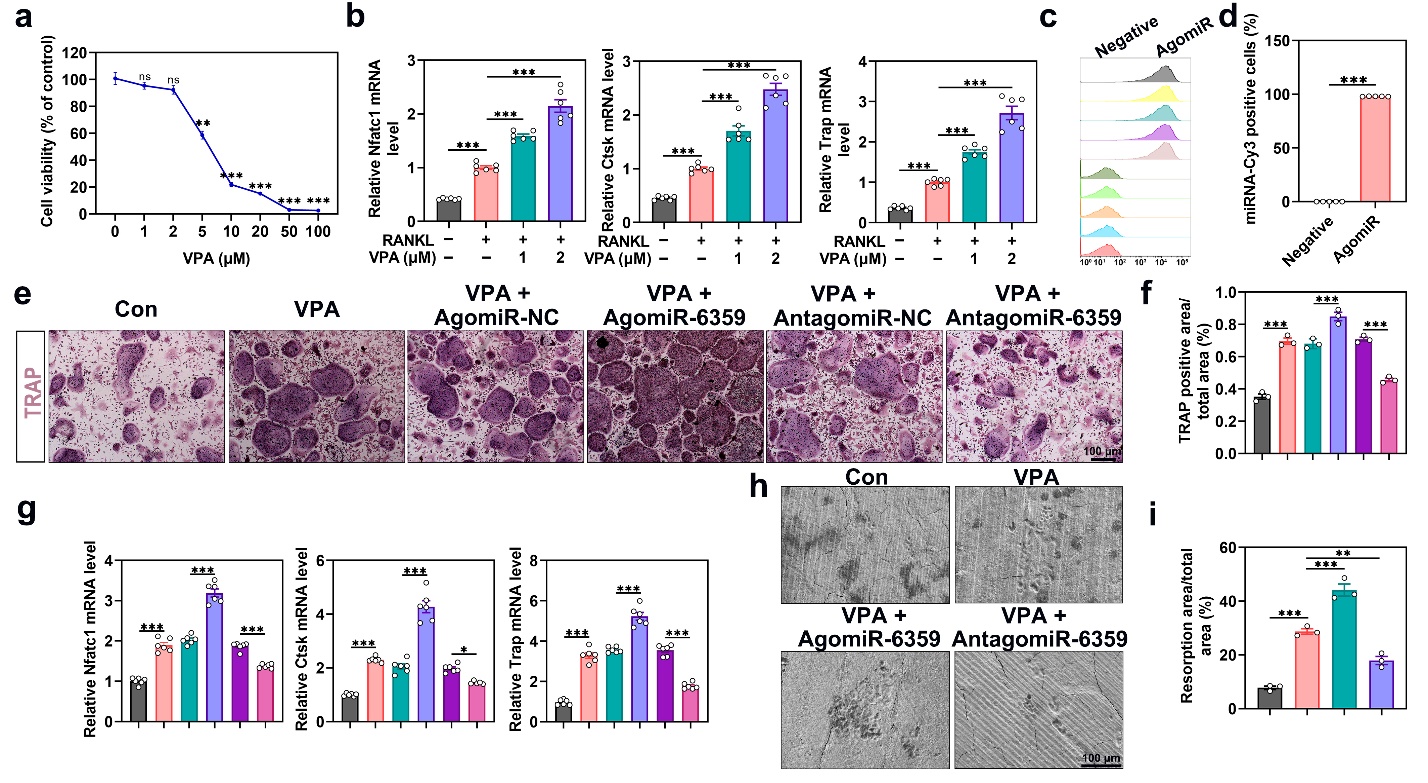
**

**Supplementary Fig 2**: (a) Cell viability was examined using the CCK8 reagent (n=3). (b) Relative mRNA levels of Nfatc1, Ctsk and Trap after treatment (n=6). (c) Flow cytometry analysis showing the presence of agomiR-Cy3 indicator in osteoclast precursors (n=5). (d) Quantification the result in (c). (e) Representative images of TRAP staining of osteoclasts following corresponding treatments (n=3). Scar bar: 100 μm. (f) Quantification of osteoclastogenesis based on TRAP staining. (g) Relative mRNA levels of Nfatc1, Ctsk, Trap after indicated treatments (n=6). (h. i) Formation of bone resorption pits (h) and quantification of the resorption area of pits (i) (n=3). Scar bar: 100 μm. All results are displayed as means ± SEM (n≥3) (**p*<0.05; ***p*<0.01; ****p*<0.001).

**
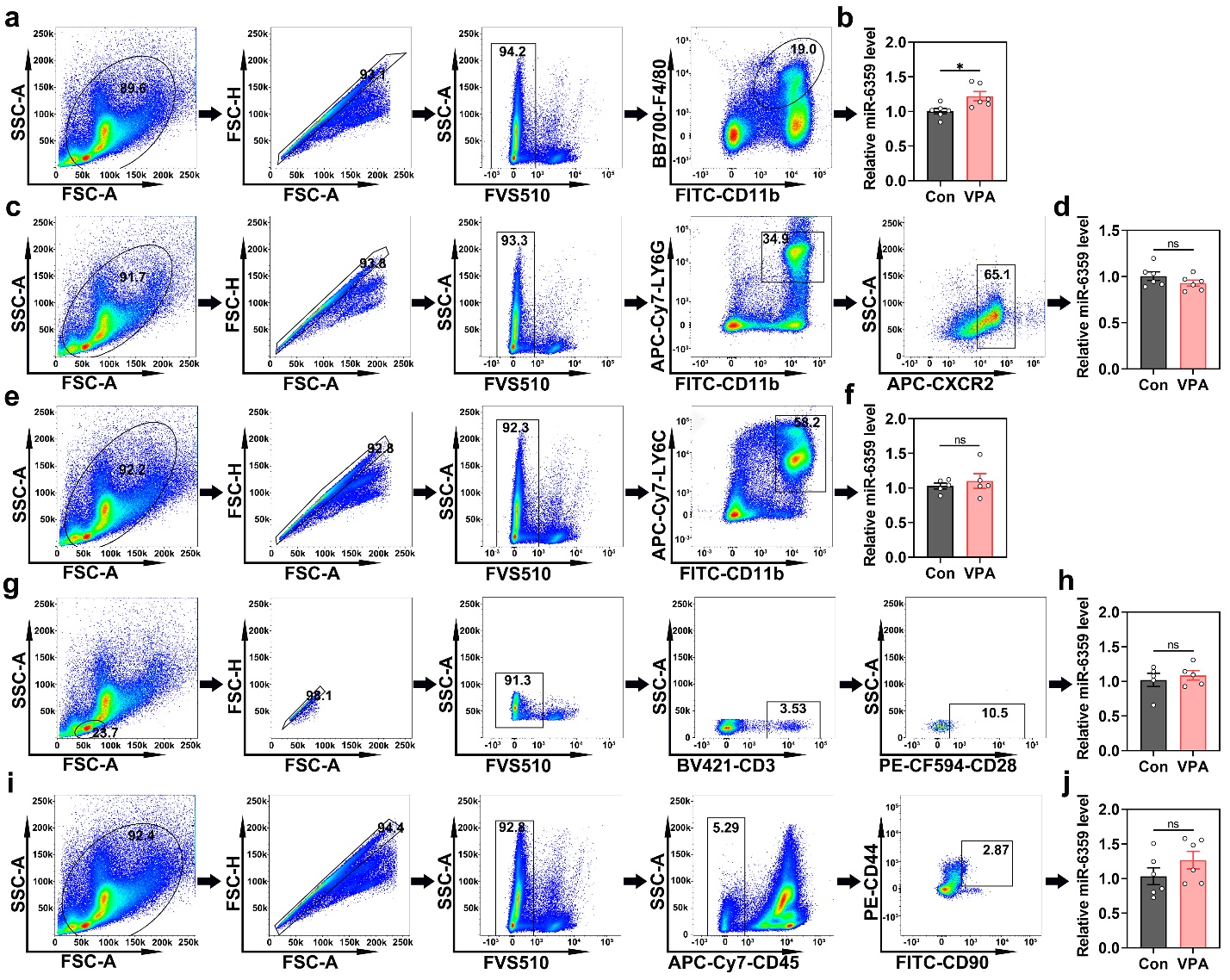
**

**Supplementary Fig 3**: (a, b) CD11b^+^, F4/80^+^ macrophages were identified (a) and the miR-6359 level was tested by qRT-PCR analysis (b) (n=6). (c, d) Flow sorting scheme for isolation of neutrophils (CD11b^+^, LY6G^+^, CXCR2^+^) from bone marrow and the expression level of miR-6359 was subsequently detected by qRT-PCR (n=6). (e, f) Representative images showed flow cytometric sorting was proceeded to isolate the monocytes (CD11b^+^, LY6C^+^) (e) and then qRT-PCR analysis was performed (f) (n=5). (g, h) The gating strategy of T lymphocytes (CD3^+^, CD28^+^) from bone marrow by the flow-cytometric analysis (g) and the expression of miR-6359 in T lymphocytes was tested (h) (n=5). (i, j) CD45^-^, CD44^+^, CD90^+^ cells (BMSCs) were sorted from bone marrow (i) and the expression of miR-6359 in sorted cells was analyzed by qRT-PCR analysis (j) (n=5). All results are displayed as means ± SEM (n≥3) (**p*<0.05; ***p*<0.01; ****p*<0.001).

**
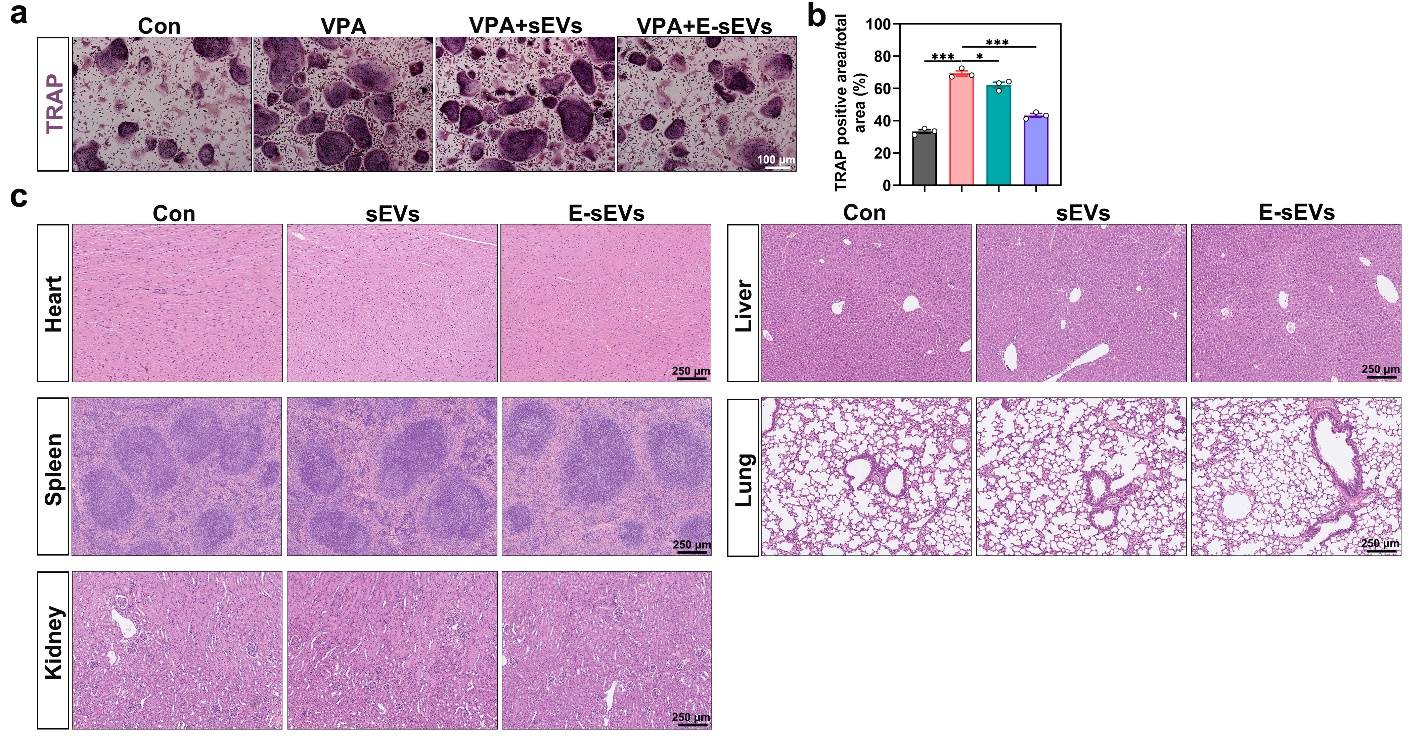
**

**Supplementary Fig 4**: (a) Osteoclast precursors were cultured with corresponding treatments. At the end of day 5, osteoclast precursors-induced cells were fixed, stained for TRAP, and observed under a light microscope (n=3). Scar bar: 100 μm. (b) The area of TRAP-positive cells/total area (%) was counted. (c) H&E staining of heart, liver, spleen, lung and kidneys (n=3). Scar bar: 250 μm. All results are displayed as means ± SEM (n≥3) (**p*<0.05; ***p*<0.01; ****p*<0.001).

**
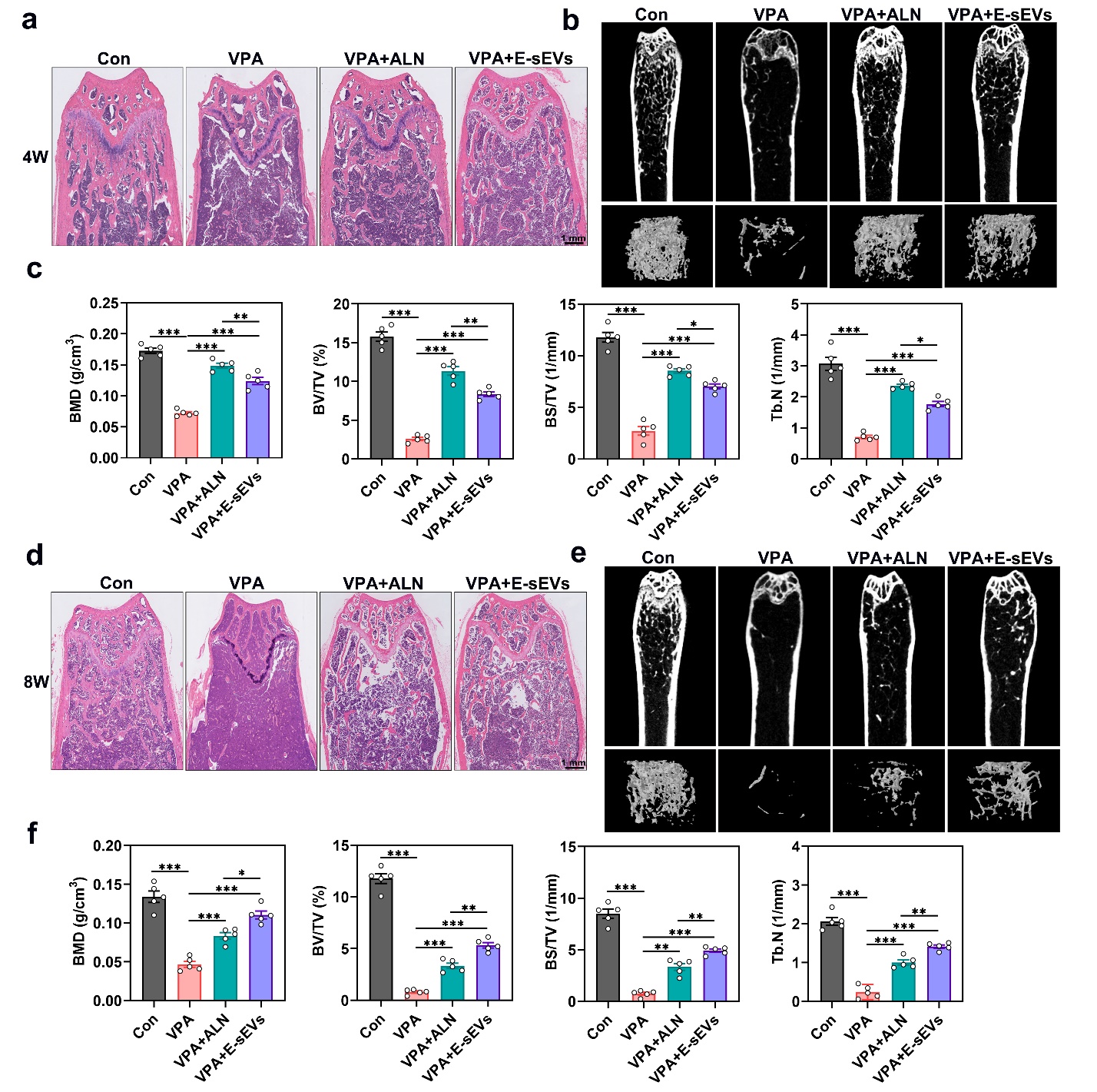
**

**Supplementary Fig 5**: (a) Representative gross pathology (H&E staining) of femurs sections from mice in control, VPA (4 weeks), VPA (4 weeks)+Alendronate (ALN) and VPA (4 weeks)+E-sEVs groups (n=5). Scar bar: 1 mm. (b) Representative micro-CT images showing bone microstructure in the distal part of femur from mice with corresponding treatments (n=5). (c) Quantification for (b) showing BMD, BV/TV, BS/TV and Tb.N. (d) Histological images of H&E staining in femurs from mice treated with vehicle, VPA (8 weeks), VPA (8 weeks) in combination with ALN or E-sEVs (n=5). Scar bar: 1 mm. (e) Representative images of micro-CT reconstruction of femurs (n=5). (f) Quantitative micro-CT analysis in (e). All results are displayed as means ± SEM (n≥3) (**p*<0.05; ***p*<0.01; ****p*<0.001).

**
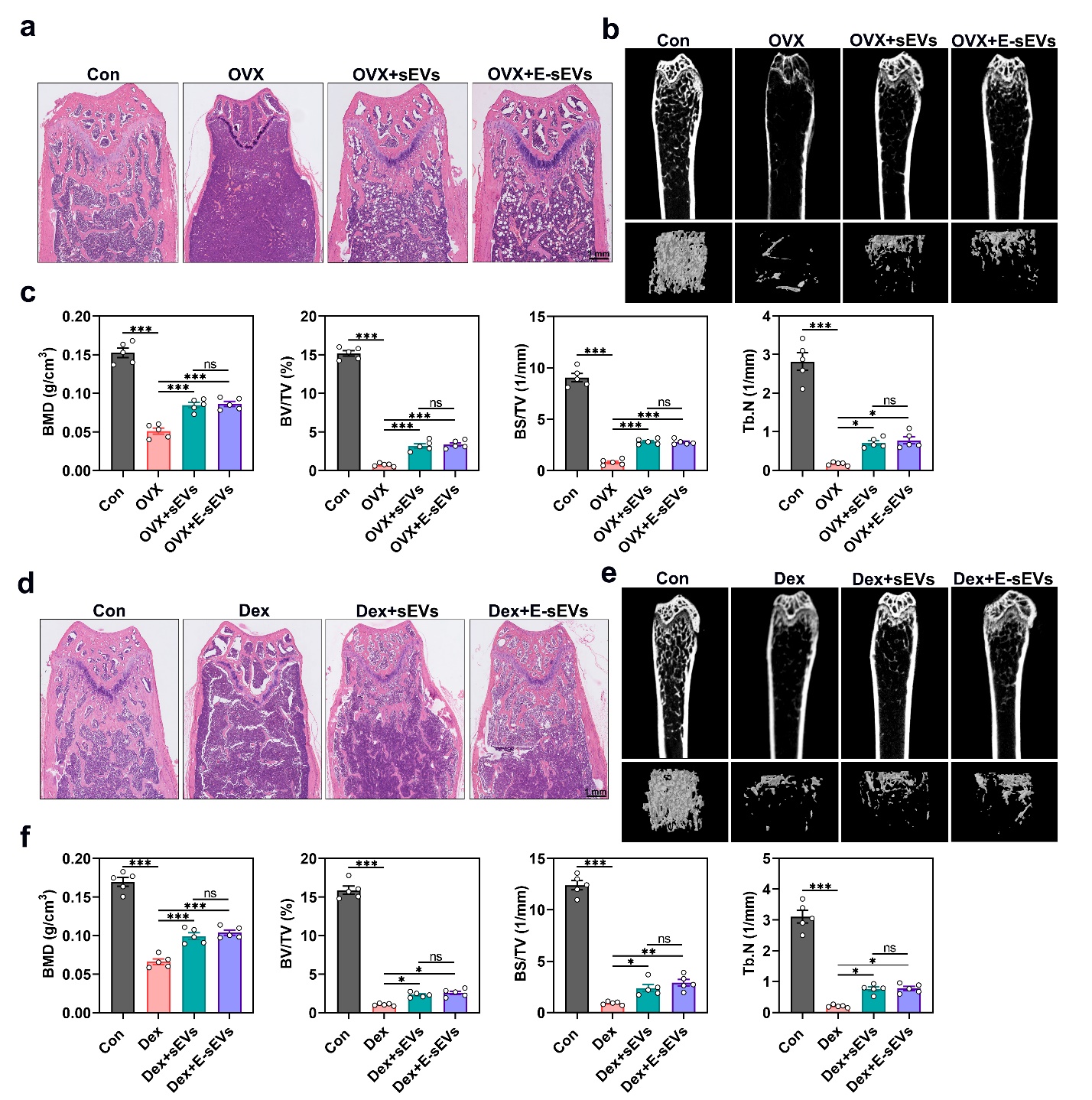
**

**Supplementary Fig 6**: (a) Representative images of H&E staining in femurs in control, OVX, OVX in combination with sEVs or E-sEVs treatment (n=5). Scar bar: 1 mm. (b) Representative micro-CT reconstruction images of femur from mice following corresponding treatments. (c) Micro-CT analysis of femurs: BMD, BV/TV, BS/TV and Tb.N (n=5). (d) Representative H&E staining images of femurs sections from mice with control, dexamethasone (Dex), Dex in combination with sEVs or E-sEVs treatment (n=5). Scar bar: 1 mm. (e) Representative micro-CT reconstruction images of femurs trabecular bones from respective groups (n=5). (f) Micro-CT analysis parameters of distal femurs: BMD, BV/TV, BS/TV and Tb.N. All results are displayed as means ± SEM (n≥3) (**p*<0.05; ***p*<0.01; ****p*<0.001).

**Supplementary Table 1: Sample information included in this study**

| **Sample** | **Gender** | **Age** | **Main condition** | **Drugs** |
| --- | --- | --- | --- | --- |
| 1 | Female | 43 | Epilepsy | Lamotrigine |
| 2 | Male | 39 | Epilepsy | Lamotrigine |
| 3 | Male | 42 | Epilepsy | Lamotrigine |
| 4 | Female | 45 | Epilepsy | Lamotrigine |
| 5 | Male | 43 | Epilepsy | Lamotrigine |
| 6 | Male | 36 | Epilepsy | Lamotrigine |
| 7 | Male | 45 | Epilepsy | Lamotrigine |
| 8 | Female | 41 | Epilepsy | Lamotrigine |
| 9 | Male | 43 | Epilepsy | Lamotrigine |
| 10 | Female | 41 | Epilepsy | Lamotrigine |
| 11 | Female | 43 | Epilepsy | VPA |
| 12 | Female | 38 | Epilepsy | VPA |
| 13 | Male | 35 | Epilepsy | VPA |
| 14 | Male | 43 | Epilepsy | VPA |
| 15 | Female | 41 | Epilepsy | VPA |
| 16 | Male | 45 | Epilepsy | VPA |
| 17 | Male | 44 | Epilepsy | VPA |
| 18 | Female | 38 | Epilepsy | VPA |
| 19 | Male | 36 | Epilepsy | VPA |
| 20 | Female | 45 | Epilepsy | VPA |

**Supplementary Table 2: Descriptive statistics for the obtained samples**

| **Group** | **Mean age (Year)** | **Duration of treatment (Year)** | **Mean time diagnose of Epilepsy** |
| --- | --- | --- | --- |
| Control group | 41.80±2.74 | 3.55±0.98 | 3.85±1.06 |
| VPA group | 40.80±3.77 | 3.0±0.75 | 3.35±0.63 |
| *P* | 0.506 | 0.176 | 0.214 |

**Supplementary Table 3：Primer sequences**

| Gene Name | Primer sequence (5’ to 3’) |
| --- | --- |
| Nfatc1-F | 5’-GGAGCGGAGAAACTTTGCG-3’ |
| Nfatc1-R | 5‘-GTGACACTAGGGGACACATAACT-3’ |
| Ctsk-F | 5’-CTCGGCGTTTAATTTGGGAGA-3’ |
| Ctsk-R | 5’-TCGAGAGGGAGGTATTCTGAGT-3’ |
| Trap-F | 5’-CACTCCCACCCTGAGATTTGT-3’ |
| Trap-R | 5’-AAGTAGTGCAGCCCGGAGTA-3’ |
| GAPDH-F | 5’-ACCCAGAAGACTGTGGATGG-3’ |
| GAPDH-R | 5’-CACATTGGGGGTAGGAACAC-3’ |
| Set-F | 5’-AGAAGAGGTCAGAATTGATCGCC-3 |
| Set-R | 5’-TGGTTGACAAATGTTGTTACCCA-3 |
| Wwtr1-F | 5’-GTGTGCCCAATGCACTGAC-3 |
| Wwtr1-R | 5’-TGACGCATCCTAATCCTCTCTC-3 |
| Sirt3-F | 5’-TACAGGCCCAATGTCACTCA-3 |
| Sirt3-R | 5’-ACAGACCGTGCATGTAGCTG-3 |
| DMD-F | 5’-GGAAAGCAACACATAGACAACCT-3 |
| DMD-R | 5’-GGGCATGAACTCTTGTAGATCC-3 |
| miR-6359-RT | 5’-GTCGTATCGACTGCAGGGTCCGAGGTATTCGCAGTCGATACGACT  TCTGA-3 |
| miR-6359- F | 5’-CCGATGTTGCCCAGGG-3 |
| U6-RT | 5’-GTCGTATCGACTGCAGGGTCCGAGGTATTCGCAGTCGATACGACAA  AAATAT-3 |
| U6-F | 5’-AGCACATATACTAAAATTGGAACGAT-3 |
| Anti-miR-6359-CGGGAGC-RT | 5’-GTCGTATCGACTGCAGGGTCCGAGGTATTCGCAGTCGATACGACG  CTCCC-3 |
| Anti-miR-6359-CGGGAGC-F | 5’-TTCTGACCCTGGGCAACATC-3 |
| MiR-138-5p-RT | 5’-GTCGTATCGACTGCAGGGTCCGAGGTATTCGCAGTCGATACGACCGGCCT-3 |
| MiR-138-5p-F | 5’-GGCAGCTGGTGTTGTGAATC-3 |
| Common-R | 5’-ACTGCAGGGTCCGAGGTATT-3’ |

**Supplementary Table 4:** **flow antibodies**

| **Antibodies** | **Dilution** | **Supplier** | **Catalog N** |
| --- | --- | --- | --- |
| FITC Mouse Anti-Rat CD90/Mouse CD90.1 | 1:200 | BD Pharmingen | 561973 |
| FITC Rat Anti-CD11b(M1/70) | 1:200 | BD Pharmingen | 557396 |
| PE Rat Anti-Mouse CD44(IM7) | 1:200 | BD Pharmingen | 553134 |
| APC-Cy7 Rat Anti-Mouse Ly-6G(1A8) | 1:200 | BD Pharmingen | 560600 |
| APC-Cy7 Rat Anti-Mouse CD45(30-F11) | 1:200 | BD Pharmingen | 557659 |
| APC Rat Anti-Mouse CD115 (CSF-1R)(T38-320) | 1:200 | BD Pharmingen | 567027 |
| BV421 Rat Anti-Mouse CD3 Molecular Complex(17A2) | 1:200 | BD Pharmingen | 564008 |
| PE/Dazzle™ 594 anti-mouse CD28 | 1:200 | Biolegend | 102124 |
| APC anti-mouse CD182 (CXCR2) | 1:200 | Biolegend | 149312 |
| BB700 Rat Anti-Mouse F4/80(T45-2342) | 1:200 | BD Pharmingen | 746070 |
| APC/Cyanine7 anti-mouse Ly-6C Antibody | 1:200 | Biolegend | 128025 |
| PE/Cyanine7 anti-mouse CX3CR1 | 1:200 | Biolegend | 149016 |
| Fixable Viability Stain 510 | 1:200 | BD Pharmingen | 564406 |
| Purified Rat Anti-Mouse CD16/CD32 (Mouse BD Fc Block)(2.4G2) | 1:200 | BD Pharmingen | 553141 |
